# Identification of cell wall synthesis inhibitors active against *Mycobacterium tuberculosis* by competitive activity-based protein profiling

**DOI:** 10.1016/j.chembiol.2021.09.002

**Published:** 2022-05-19

**Authors:** Michael Li, Hiren V. Patel, Armand B. Cognetta, Trever C. Smith, Ivy Mallick, Jean-François Cavalier, Mary L. Previti, Stéphane Canaan, Bree B. Aldridge, Benjamin F. Cravatt, Jessica C. Seeliger

**Affiliations:** 1 Department of Pharmacological Sciences and Immunology Stony Brook University, Stony Brook, NY 11790, USA; 2 Department of Microbiology and Immunology Stony Brook University, Stony Brook, NY 11790, USA; 3 Department of Chemistry, Scripps Research Institute, La Jolla, CA 92037, USA; 4 Department of Molecular Biology and Microbiology, Tufts University, Boston, MA 02111, USA; 5 Aix-Marseille Université, CNRS, LISM, IMM FR3479, 13402 Marseille, France

**Keywords:** *Mycobacterium*, tuberculosis, inhibitor, triazole urea, activity-based probe, serine hydrolase, chemoproteomics

## Abstract

The identification and validation of a small molecule’s targets is a major bottleneck in the discovery process for tuberculosis antibiotics. Activity-based protein profiling (ABPP) is an efficient tool for determining a small molecule’s targets within complex proteomes. However, how target inhibition relates to biological activity is often left unexplored. Here, we study the effects of 1,2,3-triazole ureas on *Mycobacterium tuberculosis* (*Mtb*). After screening ∼200 compounds, we focus on 4 compounds that form a structure-activity series. The compound with negligible activity reveals targets, the inhibition of which is functionally less relevant for *Mtb* growth and viability, an aspect not addressed in other ABPP studies. Biochemistry, computational docking, and morphological analysis confirms that active compounds preferentially inhibit serine hydrolases with cell wall and lipid metabolism functions and that disruption of the cell wall underlies biological activity. Our findings show that ABPP identifies the targets most likely relevant to a compound's antibacterial activity.

## Highlights

• A serine hydrolase inhibitor library is screened against *M. tuberculosis*
• Chemoproteomic profiling shows that hit compound AA692 has multiple targets
• A structure-activity series helps identify targets relevant to the mode of action
• AA692 disrupts the cell wall, likely by inhibiting lipid and cell wall biosynthesis

## Graphical abstract

**Figure:**
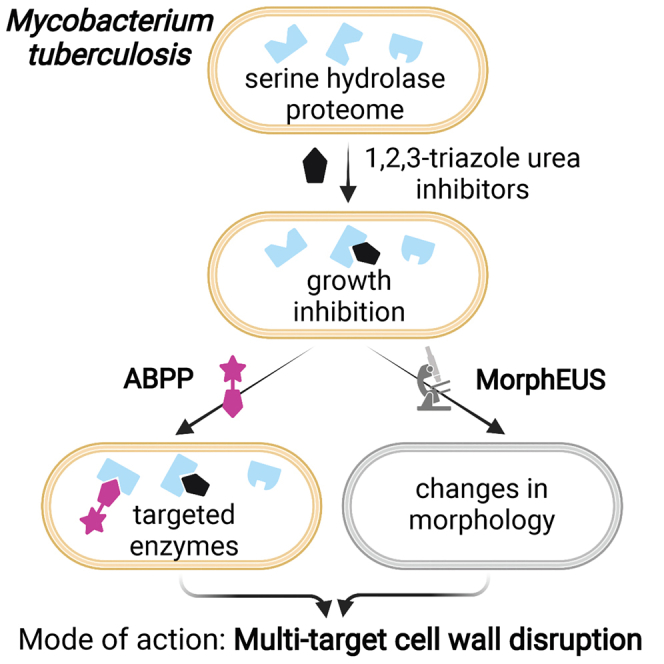

## Teaser

Identification and validation of targets are major obstacles in discovering drugs to combat *Mycobacterium tuberculosis* (*Mtb*). Here, Li et al. establish a streamlined process to identify *Mtb* inhibitors and their targets and use a structure-activity series and activity-based protein profiling to distinguish serine hydrolases relevant to inhibitor mode of action.

## Introduction

The rising incidence of antibiotic resistance in the causative bacterium *Mycobacterium tuberculosis* (*Mtb*) makes the need to develop novel tuberculosis therapies ever more urgent. *Mtb* inhibitor discovery has relied primarily on two approaches: phenotypic or target-based compound screening. In both cases, the identification and validation of a compound’s target is important for compound optimization. Target validation has depended largely on high-throughput genetic methods, such as generating spontaneous mutations (Andries et al., 2005; Christophe et al., 2009; Grzegorzewicz et al., 2012; Makarov et al., 2009; Pethe et al., 2013; Remuiñán et al., 2013; Stanley et al., 2013; Stover et al., 2000) and over- or underexpressing putative targets (Abrahams et al., 2012; Evans and Mizrahi, 2015; Johnson et al., 2019; Krieger et al., 2012; Wei et al., 2011). However, this method is less revealing when multiple targets underlie biological activity.

Indeed, inhibiting multiple targets is an important feature of some antibiotics, including the first-line tuberculosis drug isoniazid (Argyrou et al., 2006a, 2006b; Gangadharam et al., 1963; Silver, 2007). Spontaneous resistance mutations can reveal the most easily mutated targets, but not necessarily all targets relevant to compound activity. There is thus a need for a method that detects all potential targets simultaneously and thereby provides a comprehensive and accurate assessment of an inhibitor’s mode of action.

Activity-based protein profiling (ABPP) has emerged as a tool that can monitor the reactivity of nucleophiles within complex proteomes. In *Mtb*, as in other organisms, the broad utility of ABPP has inspired the modification of covalent inhibitors into probes, usually by adding an alkyne for tagging targets via azide-alkyne cycloaddition for enrichment and detection (Lehmann et al., 2016, 2018; Ravindran et al., 2014) or to investigate activity of particular enzymes (Duckworth et al., 2012; Lentz et al., 2016).

An alternative to inhibitor modification is to use ABPP competitively: inhibitor and activity-based probe (ABP) target the same reactive nucleophile, such that the inhibitor exerts its biological effects via targets detected by ABPP. In *Mtb* competitive ABPP has been used to identify, for example, the serine hydrolase (SH) targets of oxadiazolone compounds (Nguyen et al., 2018a), the cyclipostin analog CyC_17_ (Nguyen et al., 2017), and 7-urea chloroisocoumarins (Babin et al., 2021). With this strategy, enzymes related to cell wall biosynthesis and lipid metabolism were identified. However, the relative contribution of these targets to the biological activity of the inhibitors has not been determined.

In this study, we combined phenotypic screening with competitive ABPP to identify 1,2,3-triazole ureas that inhibit *Mtb* growth and their SH targets. We have previously reported 1,2,3-triazole ureas as potent and selective inhibitors of SHs through covalent inhibition of the active site serine (Adibekian et al., 2011; Hsu et al, 2013a, 2013b). We reasoned that competitive ABPP could be used to identify SH targets and also adapted to better delineate their contributions to inhibitor biological activity. We used a four-compound structure-activity series to test the hypothesis that enzymes preferentially inhibited by active versus inactive compounds more likely contribute to antibacterial activity. Biochemical assays and computational docking validated the structure-activity relationships among the selected inhibitors and supported our use of ABPP to prioritize SH targets. The functions of prioritized targets suggested activity via the inhibition of cell wall and lipid synthesis and we corroborated this finding using morphological profiling of inhibitor-treated *Mtb*.

### Experimental procedures

#### Phenotypic screening for inhibition of Mtb growth

Autoluminescent *Mtb* were grown to initial OD_600_ of 0.5–0.6 and subcultured to OD_600_ of 0.02 in modified Roisin’s medium. Where noted, 0.5% glycerol was substituted with 100 μg/mL cholesterol (from a 500× stock in 1:1 [v/v] ethanol/tyloxapol). The triazole urea library was obtained from the Cravatt laboratory (Adibekian et al., 2011). The final concentration of all compounds was 10 μM and of DMSO was 1% (v/v). Plates were incubated at 37°C with 5% CO_2_ for 7 days. Luminescence was measured with 500 ms integration time (Molecular Devices SpectraMax M3). Percent inhibition was calculated as 100 × [(μ_DMSO_ ‒ μ_Compound_)/μ_DMSO_], where μ is the average luminescence signal.

#### Target identification by ABPP-SILAC

For all experiments, *Mtb* was incubated with compounds at either 2 μM (∼1× minimum inhibitory concentration [MIC] AA691) or 13 μM (∼1× MIC AA692) and maintained at a moderate cell density to reflect conditions under which antibacterial activity was observed and to minimize clumping and promote even exposure to inhibitors. *Mtb* was cultured to OD_600_ of ∼1 in modified Roisin’s medium containing either ^14^N (“light”) or ^15^N (“heavy”) ammonium chloride as a sole nitrogen source. Light cultures were incubated with AA691, AA692, or AA702 and heavy cultures were incubated with 0.02% (v/v) DMSO vehicle control for 2 h. The cell pellet was washed with 1 mL 0.05% Tween 20 in PBS and then 2 mL PBS. Cell suspensions were lysed by bead beating at 4,000 rpm for 45 s on/off cycles (3 min total processing time; BeadBug homogenizer, Benchmark Scientific). Protein concentration in clarified lysates was measured by the BCA assay (Pierce) and lysates were diluted to 2 mg/mL with PBS in 0.5 mL total volume. Lysates were treated with 10 μM fluorophosphonate-biotin (FP-biotin) (a gift from Dr. Eranthie Weerapana) for 1 h at 22°C and desalted (PD Miditrap G-25 columns, GE Healthcare). Each light lysate was then combined 1:1 with a heavy lysate in 1 mL total volume and incubated with 2% SDS and 2 M urea final concentration in PBS in a final volume of 2 mL for 40 min at 22°C, 110 rpm. Combined lysates were spun at 4,300 × *g* for 20 min. The supernatants were diluted with 8 mL PBS and filtered twice through a 0.2-μm polyethersulfone filter. At this point the filtered lysates were deemed non-viable by viability testing for *Mtb* and removed from the Biosafety Level 3 laboratory for further analysis (see STAR Methods).

#### MorphEUS analysis

*Mtb* was cultured for 21 h at a starting OD_600_ of ∼0.7 in 7H9 broth (Thermo Fisher Scientific; DF0713-17-9) with 0.05% Tween 80 (Thermo Fisher Scientific; BP338-500), 0.2% glycerol (Thermo Fisher Scientific; G33-1), 10% Middlebrook oleic acid-albumin-dextrose-catalase (Thermo Fisher Scientific; B12351) and AA691, AA692, AA701, or AA702 at 50 or 500 μM. These concentrations correspond to ∼0.5× and 5× the MIC of AA692 under these conditions; the MIC of AA692 is higher in 7H9 (∼100 μM) than in Roisin (11 μM; Table 1). The final concentration of DMSO was 1% (v/v). Compound-treated *Mtb* cultures were fixed with 4% paraformaldehyde (Alfa Aesar, 43368) for 1 h, washed twice with 100 μL PBS containing 0.2% Tween 80 (PBST), and resuspended in 100 μL PBST. Staining and imaging were as described previously (Smith et al., 2020). In brief, 50 μL of fixed *Mtb* cells was diluted with 50 μL PBST and stained with 0.6 μg of FM4-64-FX (Thermo Fisher Scientific; F34653) and 15 μL of 0.1 μM SYTO 24 (Thermo Fisher Scientific; S7559) at room temperature in the dark for 30 min. Stained cells were washed once with 100 μL of PBST and resuspended in 30 μL of PBST. Stained *Mtb* were spotted onto agarose plates (1% [w/v] agarose; Sigma-Aldrich A3643-25G) and images were captured with a widefield DeltaVision PersonalDV (Applied Precisions) microscope. Bacteria were illuminated using an InsightSSI Solid State Illumination system with transmitted light for phase contrast microscopy and a DV Elite CMOS camera. SYTO 24 was imaged using 475 nm excitation and 525 nm emission. FM4-64-FX was imaged with 475 nm excitation and 679 nm emission. Two technical replicate images were taken from each sample for a total of 50 images per biological replicate. Three biological replicates were generated for each drug treatment.

**Table 1:** Activity of 1,2,3-triazole ureas against *Mycobacterium tuberculosis* (see also Figures S1 and S7)

Compound	Structure	Glycerol			Cholesterol	
		% inhibition^a^	MIC (μM)		% inhibition^a^	MIC (μM)
			Autoluminescence^b^	Visual inspection		Autoluminescence^b^
AA691-(2,4)		97	7 ± 2	6	98	1.7
AA692**-**(2,4)		90	12 ± 2	12	98	11
AA701		44	46 ± 18	50	32	>100
AA702		−9	>100	>100	20	>100
AA691-(1,4)			>100			
AA692-(1,4)			45			
Isoniazid		95	0.1	0.1	73	
Rifampicin		98	1	1	99	7

a Initial screen at 10 μM; average of two independent experiments.

b Average ± SD reported for n = 3–5 independent experiments; single fit or average reported for n = 1–2 independent experiments.

The morphological changes for cells treated with AA691, AA692, AA701, or AA702 were then processed and analyzed using the MorphEUS analysis pipeline and an existing reference drug set (Smith et al., 2020). The profile for each compound at a designated time point was individually applied onto the morphological space, constructed using 34 compounds with known molecular targets. Multiple classification trials (70 total) were performed for each analysis to determine the frequency of nearest neighbor connections. The resulting nearest neighbor frequency (connection strength) is highest among cells treated with drugs that target similar cellular components and pathways. This analysis therefore allows for classification of drug target(s) by determining the similarity in morphological response to drugs with known mechanisms of action.

## Results

### Triazole urea compounds inhibit the growth of *Mtb* on glycerol and on cholesterol

**Figure 1:**
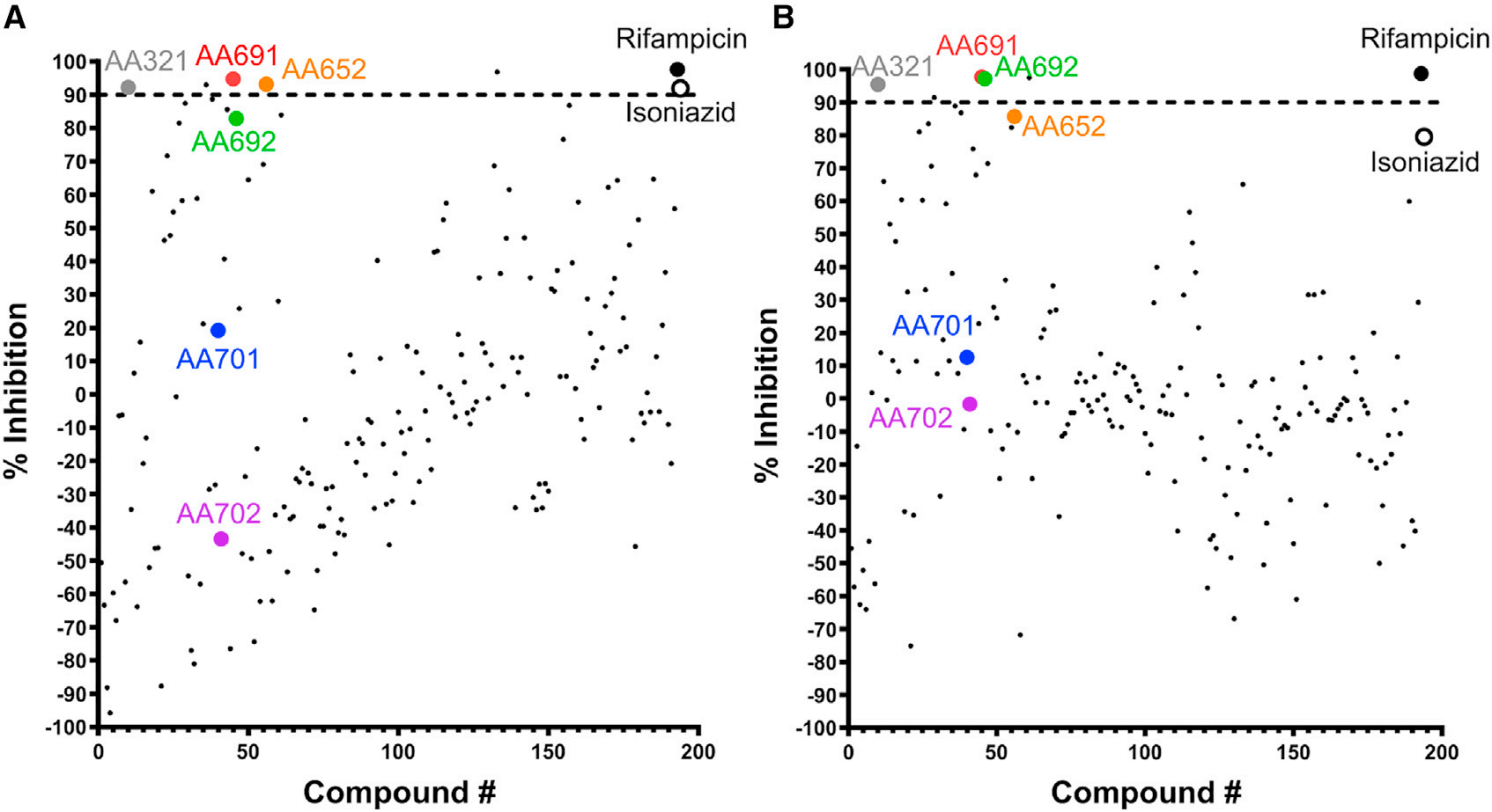
A triazole urea library yielded compounds that inhibit *Mtb* growth in both glycerol and cholesterol Autoluminescent *Mtb* was incubated for 7 days with 10 μM compound in modified Roisin’s medium with (A) glycerol or (B) cholesterol as the sole carbon source. Percent inhibition was calculated by normalizing the autoluminescence signal to DMSO vehicle-treated control. The Z′ scores were (A) 0.59 and (B) 0.49. Data shown are representative of two biological replicates.

To assess the activity of 1,2,3-triazole ureas against *Mtb*, we screened a library of 192 compounds (Adibekian et al., 2011) at 10 μM for their ability to restrict growth on glycerol and on cholesterol, a nutrient source relevant *in vivo* (Wilburn et al., 2018). We defined hit compounds as those that decreased *Mtb* autoluminescence by ≥90% versus vehicle-treated controls in both glycerol- and cholesterol-containing medium (Figure 1; Table 1). The triazole ureas AA691, AA692, AA652, and AA321 were selected as hit compounds for a hit rate of 2% (4 out of 192 compounds).

Of the four hit compounds, AA691 and AA692 were of particular interest because of their relationships to two other compounds, AA701 and AA702. In this series AA692 serves as the parent structure (Table 1). AA691 is the largest and most hydrophobic compound and differs from AA692 in the substitution of a cyclohexanol for an isopropanol group at *C4* of the substituted triazole ring. AA701 and AA702 are successively smaller and differ from AA692 at the piperidine *C4*, with a methyl or no substitution, respectively. Interestingly, AA701 and AA702 exhibited successively lower activity than AA691 and AA692 in the initial screen (44% and −9% inhibition in glycerol; 32% and 20% in cholesterol, respectively).

We verified the initial screening results by measuring the MICs for AA691, AA692, AA701, and AA702 (Figure S1). The MICs were comparable by autoluminescence and visual inspection, confirming that these compounds affect *Mtb* viability and not just the *luxABCDE* pathway. Consistent with the initial screen, AA691 and AA692 have low micromolar MICs, whereas AA701 and AA702 are ∼4- and >10-fold less active (Table 1). We also obtained the 1,4-regioisomers of AA691 and AA692, which were far less active (Table 1). For simplicity we hereafter use AA691 and AA692 to refer to the 2,4-regioisomers, except when making explicit comparisons with the 1,4-regioisomers.

### AA691 and AA692 restrict survival of replicating and non-replicating *Mtb*

**Figure 2:**
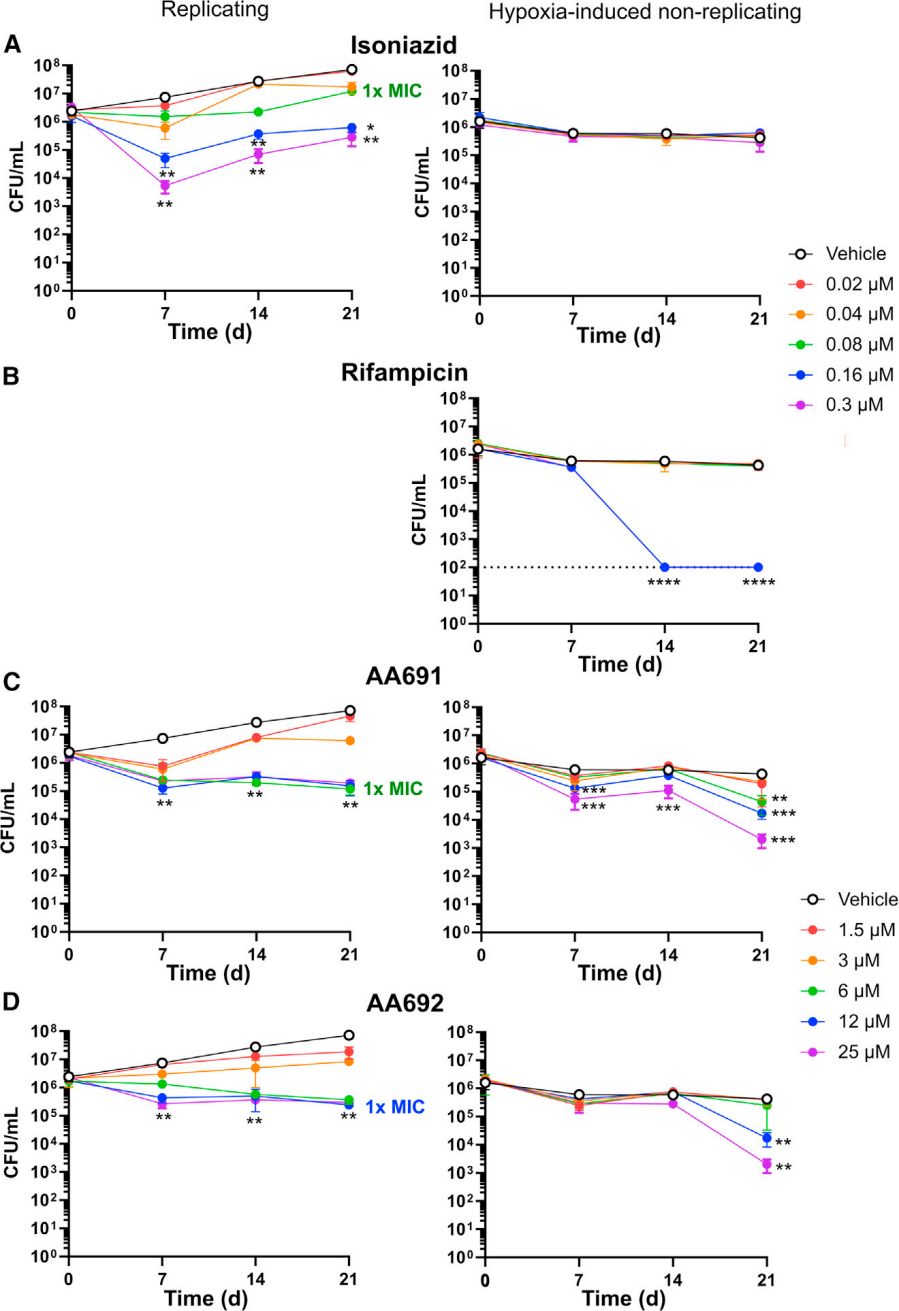
AA691 and AA692 are active against both replicating and hypoxia-induced non-replicating *Mtb* *Mtb* was treated with (A) isoniazid, (B) rifampicin, (C) AA691, or (D) AA692 in modified Roisin’s medium containing glycerol and enumerated at each time point. ∗p < 0.05, ∗∗p < 0.005, ∗∗∗p < 0.0005, ∗∗∗∗p < 0.001 by one-way ANOVA with Dunnett correction for each time point versus t = 0 (replicating) or versus vehicle control (hypoxia-induced non-replicating). Data shown are the mean ± S.D. for three biological replicates. See also Figures S2 and S3.

During human infection, *Mtb* encounters diverse environmental stressors, such as hypoxia and acidic pH (Gold and Nathan, 2017; Prosser et al., 2017). These factors can induce a state known as non-replicating persistence, in which *Mtb* remains metabolically active, grows, and divides, but does not increase in number (Rittershaus et al., 2013). Previous studies have shown that *Mtb* SHs are active under hypoxia (Ortega et al., 2016; Tallman et al., 2016) and essential for intracellular pH homeostasis in acidic pH (Vandal et al., 2008; Zhao et al., 2015), suggesting that SH inhibitors, such as AA691 and AA692, could be effective against both replicating and non-replicating *Mtb*. We cultured *Mtb* under hypoxia or in acidified medium (pH 5.0) to induce a state of non-replication (Figures 2 and S3). We then measured autoluminescence and colony-forming units (CFU) as a function of time and concentration of compound. No *Mtb* autoluminesce was detected after incubation for 1 day under hypoxia, likely because luminescence is oxygen dependent. We thus determined cell viability under hypoxia only by enumerating CFU.

Due to the slow replication of *Mtb* in modified Roisin’s medium (doubling time ∼4 days), we monitored compound activity for 21 days and confirmed that all compounds tested were stable in growth medium over this period (Figure S2). We first confirmed that the selected culture conditions led to the predicted phenotypes for *Mtb*. During exponential growth, isoniazid was bactericidal after one doubling time, as expected (Figure 2A). Recovery of growth at later time points likely reflects acquisition of isoniazid resistance as documented previously (Vilchèze and Jacobs, 2019). In contrast, the number of viable *Mtb* did not increase under hypoxia and isoniazid was not bactericidal at the same concentrations, confirming non-replication of *Mtb* and increased tolerance to isoniazid. As a positive control, we confirmed that rifampicin remains bactericidal (Figure 2B). These data are consistent with the model of hypoxia we used, in which oxygen is rapidly depleted and the *dosR* transcriptional marker of hypoxia is induced within hours (Eoh and Rhee, 2013), indicating a rapid transition into the non-replicating state.

AA691 and AA692 were bacteriostatic against replicating *Mtb*. At their respective MICs, both AA691 and AA692 significantly decreased *Mtb* viability by 7 days and by up to ∼1 log after 21 days, but this effect was not dose dependent (Figures 2C and 2D). When applied to *Mtb* just before inducing a non-replicative state by hypoxia, AA691 and AA692 had a more pronounced dose-dependent bactericidal effect with a 1-log decrease at 2× MIC and a 2-log decrease at 4× MIC compared with vehicle-treated controls after 21 days (Figures 2C and 2D). The activity of both compounds appeared to vary with time, with apparent stasis between days 7 and 14. The origin of these kinetics is not obvious and requires further investigation, although it may be related to slower metabolic responses in this hypoxia model (Eoh and Rhee, 2013). AA691 and AA692 were also bactericidal against *Mtb* pre-adapted to acidic pH, resulting in a significant decrease in *Mtb* autoluminescence by severalfold compared with vehicle-treated cells by day 11, while isoniazid activity was weaker but still significant (Figures S2D and S2E). In contrast, CFU counts showed no significant activity due to either isoniazid or AA692 at 1× MIC after 21 days, possibly due to innate variability in CFU assays that may have obscured the small (<1 log) but significant effect of AA692 revealed by autoluminescence (Figure S2F).

### AA691 and AA692 have low selectivity for *Mtb* over HepG2 human cells

The activity of AA691 and AA692 under hypoxic and acidic conditions suggested their potential use in infection models; however, AA692 is known to be toxic to murine T cells (Adibekian et al., 2010). We observed that AA691 and AA692 also exhibit biphasic cytotoxicity to HepG2 human hepatic cells at micromolar concentrations. AA691 and AA692 thus have far lower selective indices (CC_50_/MIC_90_) than isoniazid (Figure S3). While this may preclude the use of AA691 and AA692 in infection models, their low micromolar MICs against *Mtb* and the availability of a structure-activity series including AA701 and AA702 motivated our further efforts to identify targets around which more selective activity could be optimized.

### Frequency of spontaneous resistance to AA691 and AA692 is low

Genetic mutations that confer resistance to AA691 and AA692 could indicate proteins involved in the mechanism of action. However, no colonies were obtained after plating up to 4 × 10^8^ CFU *Mtb* on 5× or 10× MIC of AA691 or AA692, suggesting that the frequency of resistance is lower than ∼1 × 10^−8^. These experiments demonstrated that, under the tested conditions, the spontaneous rate of resistance to AA691 and AA692 compares favorably with that of isoniazid, for which the rate of resistance under the same conditions was >1 × 10^−6^.

### ABPP with FP-biotin identifies a core active SH proteome shared across multiple studies

We pursued a biochemical approach to identify the targets of selected inhibitors. In a previous study we used competitive ABPP with stable isotope labeling of amino acids in culture (ABPP-SILAC) in mammalian cells to identify the SH targets of triazole ureas by quantitative mass spectrometry (Adibekian et al., 2011). Here, we applied an analogous ABPP-SILAC approach to *Mtb* using the FP-biotin probe to detect the active SH proteome (Table S1). We detected a total of 105 proteins by ABPP-SILAC (across 10 samples in 4 independent experiments; Table S2), 56 of these reproducibly (Table S3). All proteins detected at pH 5.0 were also detected at pH 6.6, suggesting that the active SH profiles are similar under both conditions.

Among the 56 proteins in our active SH proteome, 48 were detected in 3 other ABPP studies in *Mtb* (Figure S4) (Babin et al., 2021; Ortega et al., 2016; Tallman et al., 2016), supporting our cutoff for SH annotation. Only two proteins were not predicted as SHs based on Pfam annotation: the putative aldehyde dehydrogenase Rv0458 and the fatty acid-CoA ligase FadD2, which are unlikely SHs due to their high homology to enzymes that do not use serine-mediated catalysis. Three predicted oxidoreductases (Rv3368c, Rv0927c, Rv2766c) are likely common contaminants in the affinity enrichment since they were not detected when comparing ABP treatment to vehicle control (experiment 1, Table S1). The remaining seven proteins that we detected were mostly hypothetical conserved proteins of unknown function, but which have been bioinformatically annotated as SHs. Similar to Ortega et al. (2016) we found that the detected SH proteome is enriched relative to the *Mtb* genome in the functional categories of lipid metabolism (16% versus 6%) and intermediate metabolism and respiration (46% versus 22%). In summary, the use of the fluorophosponate probe has identified ∼70 active SHs, 41 of which constitute a “core” proteome detected in at least 3 studies. These results suggest that variations in ABP structure and experimental procedures result in distinct, but overlapping, inventories of active SHs in replicating *Mtb*.

### Preferential inhibition of individual SHs by AA692 over AA702 indicates high-priority targets

To quantify SH inhibition by triazole ureas, we analyzed the competitive ABPP-SILAC experiments conducted at both pH 6.6 and 5.0, comparing compound- and vehicle-treated *Mtb*. We hypothesized that enzymes preferentially inhibited by AA691 and AA692 versus the ∼10-fold less active AA702 are more likely specific targets involved in the mechanism of action. Notably, AA691 was the more promiscuous inhibitor at pH 6.6 (Table S3), suggesting that AA692, as the more specific inhibitor, would better illustrate the antibacterial activity of these compounds.

To delineate key targets involved in growth inhibition by AA692, we compared the difference in percent inhibition by AA692 versus the inactive compound AA702 for each detected SH (Table S4). This analysis yielded 11 (pH 6.6) and 15 (pH 5.0) prioritized targets, 8 of which overlapped (Table 2). This high degree of overlap suggests that AA692 has both antibacterial activity and selectivity under both conditions. Most targets common to both conditions were predicted as essential and detected as active under hypoxia. We therefore focused on the 11 prioritized targets identified at pH 6.6 as those most likely to be relevant to the antibacterial activity of AA692. Four have known or predicted functions in mycomembrane lipid biosynthesis: the mycolyltransferases FbpA and FbpB (Belisle et al., 1997); the thioesterase TesA (Alibaud et al., 2011; Chavadi et al., 2011); and the lipase Rv3802c (Parker et al., 2009). In addition, the predicted penicillin-binding protein Rv1730c likely maintains peptidoglycan and thus sustains cell wall integrity.

**Table 2:** Prioritized serine hydrolase targets of AA692 identified by competitive activity-based protein profiling. See also Figure S4 and Table S1. Summary of ABPP-SILAC experiments, related to Table 2, Table S2. Peptides detected in ABPP-SILAC experiments, related to Table 2, Table S3. Active serine hydrolase proteome, related to Table 2, Table S4. Prioritized serine hydrolase targets of AA692, related to Table 2, Table S5. Visual MIC for Mtb::ribo-TesA, related to Table 2.

Protein name	Rv identifier	Function or annotation	Prioritized targets^a^					Identified as a target of			
			pH 6.6	pH 5.0	*In vitro* essential	*In vivo* essential^6^	Active under hypoxia	EZ120^10^	Lalistat^10^	CyC_17_^11^	THL^12^
**AmiB2**	Rv1263	putative amidase	●	●			●^7,8^			●	
**Rv1730c**	Rv1730c	possible penicillin-binding protein	●		●^1^				●	●	
**LipC**	Rv0220	esterase	●	●							
**Rv3591c**	Rv3591c	probable hydrolase	●	●		●	●^7^				
**FbpA**	Rv3804c	mycolyltransferase	●	●	●^1,5^	●	●^7^	●		●	
**Rv3802c**	Rv3802c	lipase	●		●^1,2,3,4^	●	●^8^				
**FpbB**	Rv1886c	mycolyltransferase	●	●			●^7^	●			
**Rv2627c**	Rv2627c	alpha-beta hydrolase	●	●							
**TesA**	Rv2928	thioesterase	●	●	●^5^	●	●^7,8,9^	●		●	●
**LipM**	Rv2284	probable hydrolase	●	●			●^7,8,9^	●	●	●	●
**Cut2**	Rv2301	probable carboxylesterase	●								
LipE	Rv3775	probable lipase		●						●	
LipG	Rv0646c	probable lipase		●		●					
LipW	Rv0217c	probable esterase		●							
Rv2854	Rv2854	probable lipase		●							
LipN	Rv2970c	carboxylic ester hydrolase		●			●^7,8^		●	●	
LipD	Rv1923	probable lipase		●							
BpoC	Rv0554	putative non-heme bromoperoxidase		●						●	

^1^Griffin et al. (2011), ^2^Sassetti et al. (2003), ^3^DeJesus et al. (2017), ^4^Xu et al. (2017), ^5^FLUTE (orca2.tamu.edu/U19/), ^6^Zhang et al. (2013), ^7^Ortega et al. (2016), ^8^Tallman et al. (2016), ^9^Ravindran et al. (2014), ^10^Lehmann et al. (2016), ^11^Nguyen et al. (2017), ^12^Ravindran et al. (2014).

a Listed in order of decreasing Δ(% inhibition), AA692-AA702 (see Table S4).

Among the other prioritized targets, Rv2627c is an uncharacterized protein; Rv3591c a possible hydrolase; LipC and LipM are esterases (Shen et al., 2012; Tallman et al., 2016) belonging to the hormone-sensitive lipase (HSL) family member proteins (i.e., Lip-HSL); AmiB2 is a probable amidase (a broad family that includes peptidoglycan-processing enzymes); and Cut2 (also known as Culp2) is a cutinase-like protein with *in vitro* esterase/phosopholipase activity (West et al., 2009). Together these findings led to our hypothesis that AA692 has antibacterial activity by inhibiting several key serine enzymes involved in cell wall biosynthesis.

### Biochemical assays and an *in silico* molecular docking study validate ABPP-SILAC target identification and structure-activity relationships

To validate the ABPP-SILAC results, we next assessed inhibitor activity *in vitro* with several purified SHs.

**Figure 3:**
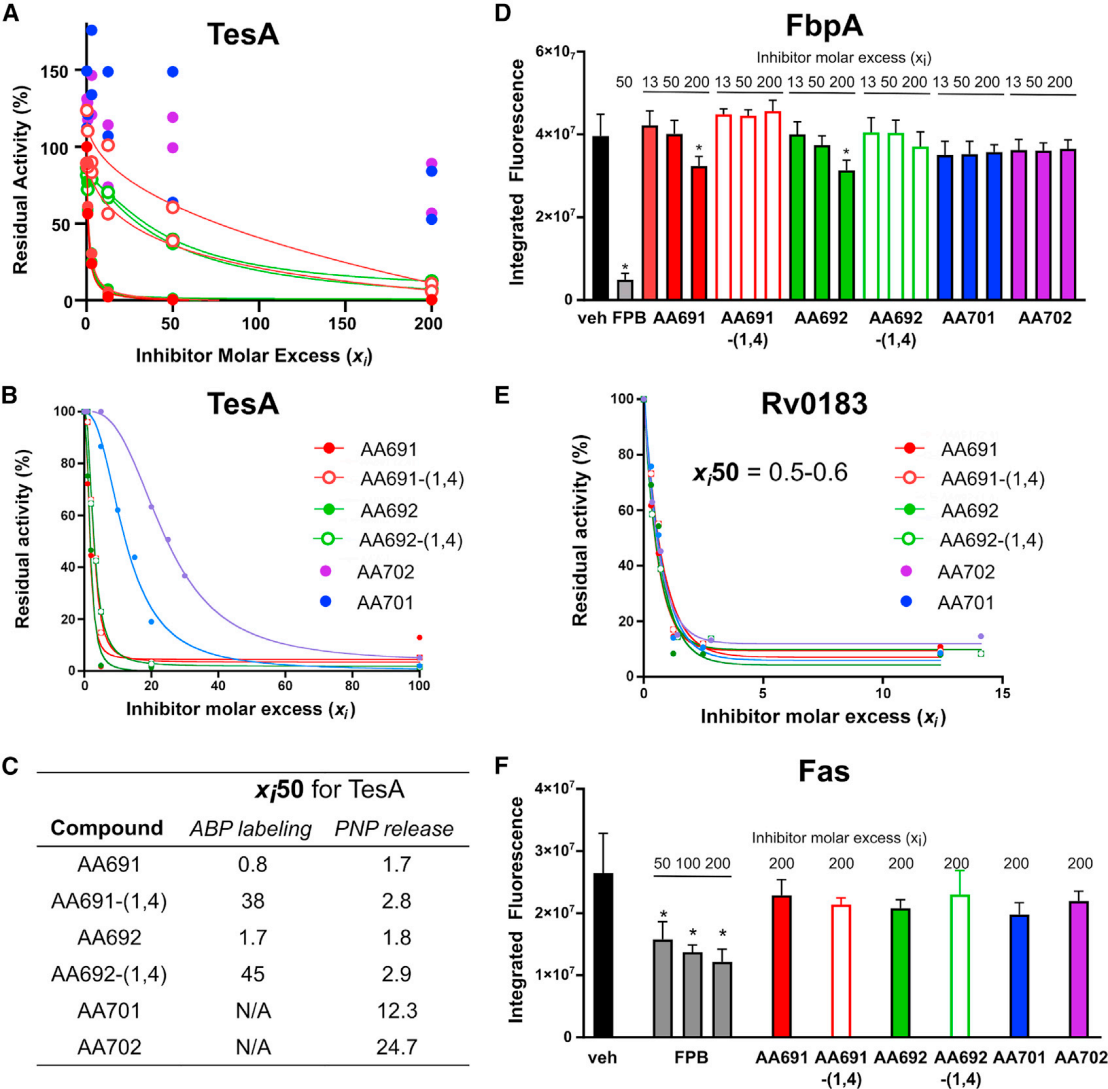
Inhibition of individual serine hydrolases recapitulates structure-activity relationships reported by competitive ABPP Residual activity of purified serine hydrolases after incubation with the indicated compounds was determined for (A–C) TesA, (D) FbpA, (E) Rv0183, and (F) Fas. The molar excess needed to reduce activity by 50% (*x*_I50_) was calculated by fitting the inhibition curve. Data shown are two independent experiments (A) (TesA) or the average ± SD of three independent experiments (all others). ∗p < 0.05 by one-way ANOVA with Dunnett correction for a given concentration versus vehicle-treated control.

Purified TesA was preincubated with each compound at various inhibitor molar excesses (*x*_I_) and then subjected to either a substrate hydrolysis assay or a competitive ABP assay with TAMRA-FP, a fluorescent ABP (Patricelli et al., 2001). A value of *x*_I50_ of 0.5 indicates a 1:1 stoichiometric ratio between the inhibitor and the lipolytic enzyme and the highest level of inhibitory activity that can be achieved. In both assays, AA691 and AA692 inhibited TesA in close stoichiometry (*x*_I50_ 0.8–1.8), in contrast with AA701 and AA702, which both exhibited >5-fold higher *x*_I50_ and thus lower relative inhibition (Figures 3A–3C). We then used the competitive ABP assay to characterize the inhibition of FbpA with similar results (Figure 3D). The regioisomers AA691-(1,4) and AA692-(1,4) showed intermediate potency against both enzymes (Figures 3C and 3D). In contrast, all tested compounds fully impaired the activity of lipase Rv0183, which by ABPP-SILAC was inhibited >95% by all compounds (Figure 3E; Table S3).

**Figure 4:**
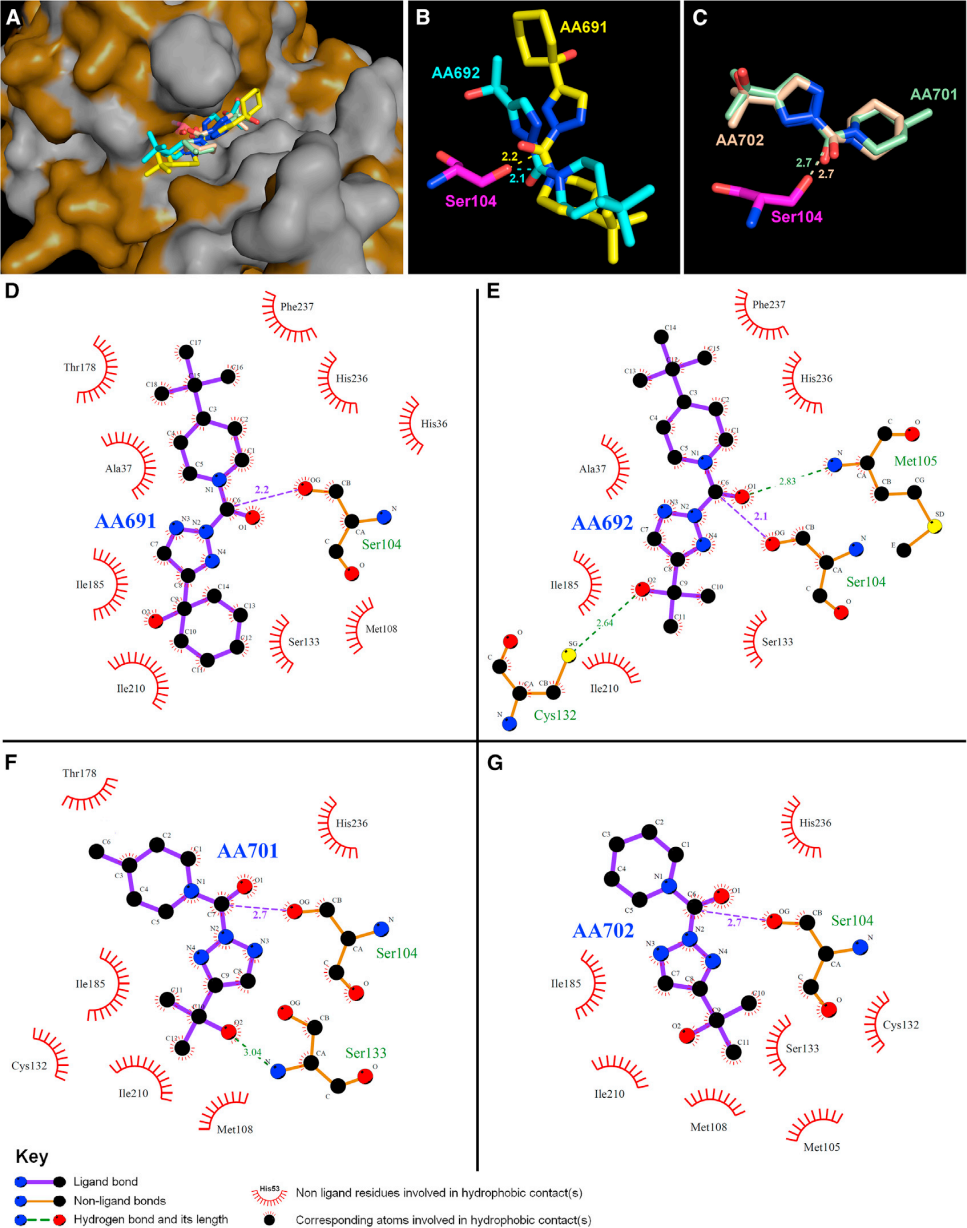
AA691 and AA692 make more contacts and are positioned closer to the catalytic serine than AA701 and AA702 in the TesA active site (A) *In silico* molecular docking of AA691, AA692, AA701, and AA702 into the crystallographic structure of TesA in a van der Waals surface representation. Hydrophobic residues are highlighted in white. Superimposition of the top-scoring docking position of (B) AA691 (yellow) and AA692 (cyan) and (C) AA701 (pale green) and AA702 (wheat) in the vicinity of the catalytic Ser104 (magenta). Ligplot^+^ (Laskowski and Swindells, 2011) analyses showing the ligand-protein interactions for (D) AA691, (E) AA692, (F) AA701, and (H) AA702 in the TesA active site with hydrogen bonds (purple, green dashed lines) and hydrophobic interactions (red) indicated. See also Figure S5.

We next used *in silico* docking to examine the predicted binding modes of AA691, AA692, AA701, and AA702 in the active sites of TesA and Rv0183. In these models AA691 and AA692 occupy the entire active site crevice of TesA and the carbonyl is at a favorable distance (2.1–2.2 Å) and orientation for forming a covalent bond with the catalytic Ser104 (Figures 4A and 4B). AA701 and AA702 adopt a similar binding mode when docked, but are farther from Ser104 (2.7 Å) (Figure 4B). AA691 obtained the most favorable binding interaction; AA702 achieved the least favorable (Δ*E* = −7.0, −6.7, −6.0, and −5.7 kcal/mol for AA691, AA692, AA701, and AA702, respectively). AA691 and AA692 are stabilized by an overlapping set of hydrophobic interactions (with His36, Ala37, Met108, Ser133, Thr178, Ile185, Ile210, His236, and Phe237; those unique to AA691 are underlined; Figures 4D and 4E). Binding of AA692 is also supplemented by two hydrogen bonds with Met105 and Cys132. Finally, the poses of AA701 and AA702 are almost superimposable, with a hydrogen bond to Ser133 for AA701 and similar hydrophobic interactions, but fewer contacts overall than for AA691 or AAA692 (Figures 4F and 4G).

In contrast, the computationally predicted binding modes for the compounds in Rv0183 were far more similar. All were predicted to adopt comparably productive orientations inside the enzyme active site (Figures S5A–S5C), with similar distances from the catalytic Ser110 (<2.5 Å) and similar predicted binding energy values (Δ*E* = −7.3 to −7.5 kcal/mol). Also, each inhibitor would be stabilized by largely the same hydrophobic interactions and hydrogen bonds (Figures S5D–S5G). Together, the predicted binding orientations, energies, and interactions in TesA and Rv0183 corroborate the biochemical inhibition data and the relative inhibitory potencies of the four inhibitors.

Given our hypothesis that AA692 disrupts lipid and cell wall metabolism, we also purified Fas, an essential fatty acid synthase that was detected in two previous studies (Ortega et al., 2016; Tallman et al., 2016), but not ours. None of the compounds inhibited Fas significantly in the competitive ABP assay (Figure 3F). Competition by biotin-FP was weak, showing that this probe does not efficiently label Fas and potentially explaining why Fas was not detected in our ABP profile.

### Overproduction of TesA does not significantly alter sensitivity to AA691 or AA692

We hypothesized that modulating the expression of targets involved in the mode of action would lead to corresponding changes in MIC. Inducible overproduction of TesA in *Mtb* led to a slight, 2-fold increase in the MIC of AA691 and AA692, but in only one of two experimental replicates (Table S5). Overproduction of single targets, including TesA, has not significantly modulated sensitivity to other SH inhibitors (Nguyen et al., 2017; Ravindran et al., 2014), supporting multi-target inhibition as integral to the activity of SH inhibitors in general.

### Morphological profiling confirms that AA691 and AA692 inhibit *Mtb* growth by disrupting cell wall synthesis

**Figure 5:**
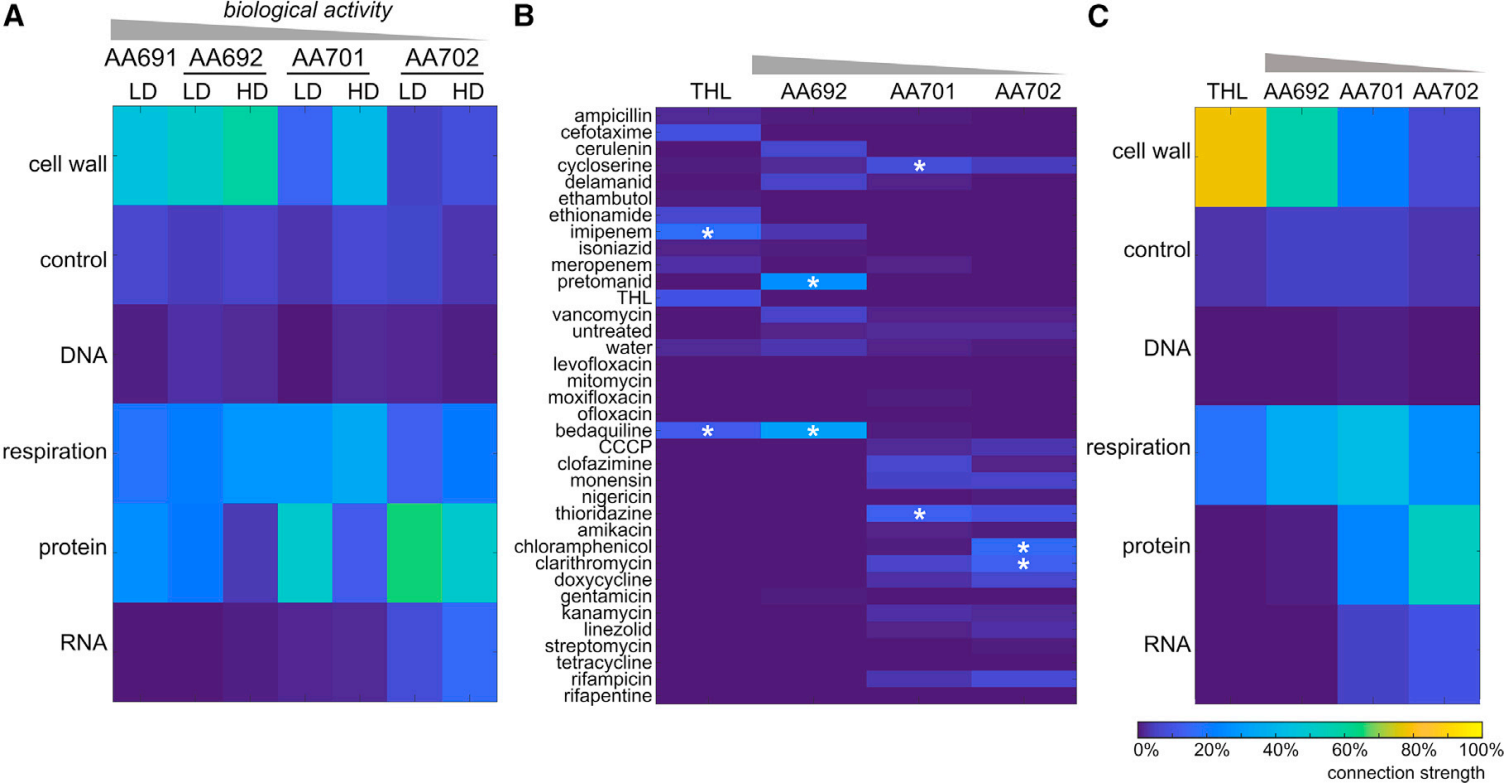
AA691 and AA692 cause morphological changes in *Mtb* similar to those induced by cell wall inhibitors *Mtb* was incubated with 50 μM (low dose [LD]) or 500 μM (high dose [HD]) of the designated compounds prior to imaging for morphological features. Following MorphEUS analysis of the resulting profiles, the nearest neighbor frequency (connection strength) based on (A), (C) broad categories for mode of action, or (B) individual compounds is highest among drugs that cause similar types of cellular change. (A) Shows data at both LD and HD; (B and C) are joint profiles from the simultaneous analysis of LD and HD results (except for THL, for which the HD profile is shown). Asterisks indicate two most frequent neighbors for each compound. See also Figure S6.

To better understand the mode of action underlying AA692’s antibacterial activity, we used a recently developed morphological profiling platform called MorphEUS (Smith et al., 2020). MorphEUS is based on the principle that drugs with similar mechanisms of action will induce similar changes in bacterial morphologies. Consistent with our target analysis, AA692 treatment of replicating *Mtb* caused morphological changes similar to those induced by cell wall synthesis inhibitors at both low and high dose (∼0.5× and 5× MIC for AA692; Figure 5A). This pattern was also observed for AA691 at low dose. Overall, MorphEUS analysis implicates disruption cell wall synthesis by our hit compounds AA691 and AA692 and weakly or not at all in the less active compounds AA701 and AA702.

This conclusion is further supported by reviewing the nearest neighbors of AA692 in the MorphEUS analysis: pretomanid and bedaquiline (Figure 5B). In replicating *Mtb* pretomanid has been shown to act in part by inhibiting the biosynthesis of essential mycolic acids (Stover et al., 2000). Although bedaquiline is an ATP synthesis inhibitor, we and others have shown that the resulting downstream metabolic perturbation produces morphological changes that resemble those from cell wall-acting inhibitors (Mackenzie et al., 2020; Smith et al., 2020).

For comparison we also applied MorphEUS to THL (Ravindran et al., 2014). THL shows a stronger connection to cell wall synthesis than AA692, likely because AA692 is also connected to protein translation inhibitors (Figures 5B and 5C). Protein translation may be another pathway by which AA691 and AA692 exert activity against *Mtb* since the translation inhibitor clarithromycin is among the nearest neighbors of both AA691 and AA692 at low dose (Figure S6). Alternatively, clarithromycin may have unrecognized effects on the cell wall that cause morphological effects similar to AA692. Overall, the MorphEUS analysis confirms disruption of cell wall biosynthesis as the direct consequence of AA691 and AA692 antibacterial activity, a finding that correlates well with our target analysis by competitive ABPP.

### AA691 and AA692 have narrow-spectrum antibacterial activity

Given the range of SHs targeted by all the 1,2,3-triazole ureas, we investigated whether our hit and related compounds might have activity against other bacteria. The antibacterial activity of AA691, AA692, AA701, and AA702 was assessed against *Escherichia coli*, *Staphylococcus saprophyticus*, and *M. smegmatis* as representative Gram-negative, Gram-positive, and non-pathogenic mycobacterial organisms, respectively. AA691 and AA692 inhibited *M. smegmatis* growth with MICs of ∼20 and ∼70 μM, respectively (Figure S7A). Consistent with their relative activities in *Mtb*, AA701 and AA702 had MICs >100 μM against *M. smegmatis.* In contrast, all four compounds had no detectable activity against *E. coli* or *S. saprophyticus* up to 100 μM (Figures S7B and S7C). These results support AA691 and AA692 as narrow-spectrum inhibitors.

## Discussion

In this study from a small ∼200-compound library we achieved a hit rate of 2%, higher than the <1% hit rate reported in other high-throughput anti-tuberculosis (TB) drug screens (Manjunatha and Smith, 2015). Of these hit compounds, the two that were the focus on this work, AA691 and AA692, behaved similarly to SH inhibitors such as THL (Ravindran et al., 2014), lalistat, and two cyclophostin analogs for which activity against non-replicating mycobacteria has been reported. Overall, our results provide foundational data on the potential of 1,2,3-triazole ureas to limit the survival of both replicating and drug-tolerant non-replicating *Mtb*. Moreover, competitive ABPP enabled identification of proteins preferentially targeted by the hit compound AA692 by comparison with inactive compound AA702. While this prioritization was based on single biological replicates under each condition, precluding statistical analysis of significance, the subsequent inhibition assays and *in silico* docking with individual targets validated our competitive ABPP results.

In contrast with our study, the targets of other SH inhibitors, such as THL, lalistat, EZ120, and CyC_17_, were identified without comparison with the targets of a related inactive compound (Lehmann et al., 2016, 2018; Nguyen et al., 2017; Ravindran et al., 2014). AA691 and AA692 share multiple targets with these SH inhibitors, including the conditional essential enzymes FbpA, TesA, and Rv1730c (Table 2). However, by comparing the profiles of our hit compounds against less active ones, we were able to exclude spurious target SHs identified in previous studies. Six of the 11 prioritized targets of AA692 (Rv0183, LipG, LipH, LipO, AmiC, and Rv0293c) were excluded due to a high degree of inhibition by AA702. Four of these excluded targets—Rv0183, the phospholipase/thioesterase LipG (Santucci et al., 2018), the esterase LipH (Canaan et al., 2004), and the putative lipase LipO—were also identified as direct targets of alkyne-modified lalistat, THL, or EZ120 (Lehmann et al., 2016, 2018; Ravindran et al., 2014). Inclusion of AA702 as an inactive control suggests that these enzymes are non-selectively targeted by active and inactive antibacterial compounds and that they may thus be unrelated to restricting *Mtb* growth or survival.

For the six enzymes prioritized exclusively by comparing AA692 and AA702 target profiles, their contributions to the activity of AA692 ultimately depends on the degree to which they must be inhibited to affect bacterial growth or survival. *Mtb* genome-wide data on target vulnerability using CRISPRi was published just after this paper was accepted and will inform further investigations into the specific targets that contribute to inhibitor mode of action (Bosch et al., 2021). However, target vulnerability may change when other targets are simultaneously inhibited; these probable effects have yet to be explored experimentally.

By tracking morphological changes in *Mtb* after compound treatment, we found that compounds with weak antibacterial activity closely resemble translation inhibitors, while AA691 and AA692 resemble cell wall synthesis inhibitors. Such comparisons between active and inactive antibacterial compounds are integral to narrow down the mode of action, especially with multi-target inhibitors that are refractory to resistance mutations. Overall, these results strongly support that AA691 and AA692 disrupt cell wall synthesis by inhibiting lipid metabolism and cell wall synthesis enzymes identified by ABPP. Based on both ABPP and microbiological analyses, all of the SH inhibitors characterized in mycobacteria to date likely act by inhibiting cell wall synthesis via the inhibition of multiple enzymes.

Despite the broad targeting of SHs by triazole urea compounds, AA692 exhibited appreciable antibacterial activity only against mycobacteria. Although 1,2,3-triazole ureas have been shown to inhibit SHs in live *S. aureus* (Chen et al., 2019), we favor the hypothesis that the hundreds of predicted mycobacteria SHs, many of which are annotated as essential, compared with the dozens so far detected in *E. coli* or *Staphylococcus* species inhibit SHs in live *S. aureus* (Keller et al., 2020; Shamshurin et al., 2014). Of the targets prioritized by ABPP (Table 2), only TesA, Rv1730c, and the predicted hydrolase Rv2627c, are not encoded by *M. smegmatis.* The absence of these SHs might explain the weaker effect of AA691 and AA692, although we cannot rule out other factors, such as additional targets or differences in cell envelope permeability in *M. smegmatis*. The selectivity for mycobacteria might be related to the unique composition of the mycobacterial cell envelope (Brennan and Nikaido, 1995) and the ability of small hydrophobic compounds to cross the lipid-rich cell wall, which is especially relevant given that SHs preferentially targeted by AA692 have lipid- and cell wall-related functions.

The low selectivity index for AA691 and AA692 advises against their use as preclinical candidates or in infection models of TB. However, triazole ureas specific for individual mammalian SHs have been developed (Adibekian et al., 2011; Hsu et al, 2013a, 2013b), suggesting that the same is possible in *Mtb*. The synthetic simplicity of 1,2,3-triazole ureas could facilitate library expansion into further structure-activity relationships to improve activity while lowering toxicity. Our observations support SH-targeted inhibitor libraries as well positioned to exploit these pharmacologically vulnerable enzymes.

Our results confirm a limitation of the competitive ABPP approach: The detection of targets relies on the promiscuity of the competing ABP label. Nevertheless, competitive ABPP offers ease and versatility since hit compounds do not need to be chemically modified and re-validated. Our work demonstrates the importance of matching of inhibitor libraries and probes to ensure that targets will be accurately captured by the probe. While these tools are most readily available for SHs, new ABPs are continually being developed to target additional chemistries on proteins, affording exciting new opportunities for competitive ABPP in inhibitor discovery and characterization.

## Significance

**Drug discovery efforts against *Mycobacterium tuberculosis* have been most successful by screening compound libraries in phenotypic cell-based assays. However, target deconvolution and validation remain major hurdles, especially if hit compounds inhibit multiple targets. We combined phenotypic screening with activity-based protein profiling to identify the serine hydrolase targets for 1,2,3-triazole ureas that restrict *M. tuberculosis* growth. Comparisons across a four-compound structure-activity series were essential to identifying targets relevant to hit compound activity and deprioritizing enzymes non-specifically inhibited by all compounds. Additional biochemical and morphological assays confirmed that hit compounds act via the disruption of cell wall and lipid metabolism. Our results underscore multi-target inhibition as key feature of serine hydrolase inhibitors with antimycobacterial activity.**

## STAR★Methods

### Key resources table

**Table:** 

REAGENT or RESOURCE	SOURCE	IDENTIFIER
**Bacterial and virus strains**		

*Mycobacterium tuberculosis* H37Rv	BEI Resources	Cat# NR-123
*Escherichia coli* MG1655	A gift from Dr. David Thanassi, Stony Brook University	N/A
*Staphylococcus saprophyticus*	ATCC	Cat# BAA-750
*Mycobacterium smegmatis* mc^2^155	ATCC	Cat# 700084

**Chemicals, peptides, and recombinant proteins**		

FP-biotin	A gift from Dr. Eranthie Weerapana, Boston College (Liu et al., 1999)	N/A
FP-TAMRA	A gift from Dr. Micah Niphakis, Lundbeck La Jolla Research Center (Patricelli et al., 2001)	N/A
Triazole urea library	Benjamin Cravatt Laboratory, Scripps Research (Adibekian et al., 2011)	N/A
Orlistat	Sigma	Cat# O4139
AA691-(1,4)	Pharmaron	N/A
AA692-(1,4)	Pharmaron	N/A

**Critical commercial assays**		

BacTiter-Glo	Promega	Cat# G8230

**Deposited data**		

Proteomics datasets	This paper	PRIDE: PXD026213
MorphEUS data analysis	This paper	https://gitlab.tufts.edu/tsmith13/morpheus-stonybrook-serine-hydrolase DOI: 10.5281/zenodo.5348021

**Experimental models: Organisms/strains**		

*Mycobacterium tuberculosis* H37Rv mLux	This paper	N/A
*Mycobacterium smegmatis* mc^2^155 mLux	This paper	N/A

**Recombinant DNA**		

mLux plasmid	A gift of Dr. Jeffrey Cox, University of California, Berkeley	N/A
pDEST14-His-Rv0183	Cotes et al. (2007)	N/A
pDEST14-TesA	Nguyen et al. (2018b)	N/A
pDEST14-TesA^S104A^	Nguyen et al. (2018b)	N/A
pMT100-*Strep-Flag-fas1*	Baron et al. (2018)	N/A
pACYCDuet-*Ara*-*acpS*	Baron et al. (2018)	N/A
pET-15b_6His_Ag85A	BEI Resources	Cat# NR-13292
pRiboI-TesA	This paper	N/A
omlp562	Integrated DNA Technologies	N/A
omlp559	Integrated DNA Technologies	N/A

**Software and algorithms**		

MorphEUS analysis	Smith et al. (2020)	N/A
RAWXtract 1.9.9.2	McDonald et al. (2004)	http://fields.scripps.edu/yates/wp/?page_id=17
ProLuCID	Xu et al. (2015)	http://fields.scripps.edu/yates/wp/?page_id=17
CIMAGE	Weerapana et al. (2010)	https://github.com/radusuciu/cimage-simple
Prism 8	GraphPad	N/A
Image Studio Lite	LI-COR	N/A
Pymol version 1.4	Schrödinger, LLC	N/A
Ligplot+	Laskowski and Swindells (2011)	https://www.ebi.ac.uk/thornton-srv/software/LigPlus/
AutoDock/Vina PyMOL plugin	Seeliger and de Groot (2010)	https://www3.mpibpc.mpg.de/groups/de_groot/dseelig/adplugin.html
ChemDraw	PerkinElmer	N/A
Chem3D Ultra 11.0	PerkinElmer	N/A
Avogadro	Hanwell et al. (2012)	http://avogadro.cc/

### Resource availability

#### Lead contact

Further information and requests for resources and reagents should be directed to and will be fulfilled by the lead contact, Jessica Seeliger (jessica.seeliger@stonybrook.edu).

#### Materials availability

Compounds synthesized for this study are available upon request. Strains generated for this study are available upon request.

### Experimental model and subject details

#### Bacterial strains and growth media

*Mtb* H37Rv (BEI Resources NR-123) was used for ABPP and spontaneous resistant mutagenesis experiments. *Mtb* H37Rv harboring the integrating mLux plasmid (gift of Jeffery S. Cox), which expresses a codon-optimized bacterial *luxABCDE* operon for autoluminescence, was used for inhibitor screening, minimum inhibitor concentration (MIC) determination, and colony forming unit (CFU) enumeration. Autoluminescence has been previously validated as an indicator of *Mtb* viability (Zhang et al., 2012). Autoluminescent *M. smegmatis* (ATCC 700084) harboring the same integrating mLux plasmid; *E. coli* (strain MG1655) and *S. saprophyticus* (BAA-750, ATCC) were used for MIC determination.

For initial cultures frozen stocks of *Mtb* were thawed and used to inoculate Middlebrook 7H9 medium (BD) containing 10% (v/v) oleic acid-albumin-dextrose-catalase (OADC) supplement (BD), 0.5% glycerol, and 0.05% Tyloxapol (Sigma). Cells were then pelleted at 4000 x *g* for 10 minutes and washed once with modified Roisin's medium (1 g/L KH_2_PO_4_, 2.5 g/L Na_2_HPO_4_, 5.9 g/L NH_4_Cl, 2.0 g/L K_2_SO_4_, 1.0 g/L citric acid, 0.08 mg/L ZnCl_2_, 0.4 mg/L FeCl_3_-6H_2_O, 0.02 mg/L CuSO_4_, 0.02 mg/L MnCl_2_-4H_2_O, 0.02 mg/L Na_2_B_4_O_7_-10H_2_O, 0.02 mg/L (NH_4_)_6_Mo_7_O_24_-4H_2_O, 0.5 mM CaCl_2_, 0.5 mM MgCl_2_, 0.5% glycerol, 0.5 mg/L biotin, 0.05% Tyloxapol, pH 6.6), and then resuspended in modified Roisin's medium. Acidic pH was achieved by buffering the medium to pH 5.0. In the acidic pH model, *Mtb* was cultured in pH 6.6, pelleted at 4000 x *g* for 10 minutes, washed once with pH 5.0 buffered modified Roisin's medium and incubated at pH 5.0 for 3 days as an adaptation step before further experimentation. *Mtb* cultures were incubated with shaking at 110 rpm or without shaking if in 96-well plates. *M. smegmatis* was inoculated from frozen stocks and cultured in modified Roisin's medium. *E. coli* and *S. saprophyticus* were similarly cultured in LB medium. All bacteria were cultured at 37°C.

### Method details

#### Minimum inhibitory concentration (MIC) determination

Autoluminescent *Mtb* was subjected to the phenotypic screening protocol except compound concentrations varied from 100 μM to 0.2 μM. Autoluminescent *M. smegmatis* was subjected to the same protocol except incubation time was 10 hours (∼3 doubling times) and the signal was measured with 400 ms integration time using a FilterMax F5 (Molecular Devices). *E. coli and S. saprophyticus* were cultured by an analogous procedure except that the incubation time was adjusted to ∼3 doubling times for the respective bacterium (1.5 hours and 2 hours respectively). For *M. smegmatis* viability was measured by adding 100 μL BacTiter-Glo (Promega) to each well and incubating at 22°C for 2 minutes before measuring luminescence as above. The linear range for each bacterium was determined by calibrating luminescence signal against OD_600_. MIC_90_ was determined by fitting the percent inhibition (versus DMSO vehicle-treated control) as a function of compound concentration to the Gompertz equation in GraphPad Prism 8. An estimated MIC_90_ by visual inspection was determined by the same protocol, but in clear round-bottom 96-well plates. For this method the lowest concentration of compound at which no visible growth was observed was reported as the MIC_90_.

#### Compound toxicity

Synthesis of AA691-(1,4) and AA692-(1,4) and toxicity experiments were performed by Pharmaron. HepG2 cells were initially cultured in Dulbecco's Modified Eagle's Medium supplemented with 10% FBS (DMEM/FBS), 1x penicillin-streptomycin mixture and 1x non-essential amino acids. Medium was aspirated, 3 mL trypsin/EDTA solution was added, and cells were incubated at 37°C for approximately 2 minutes or until the cells were detached and floated. Trypsin/EDTA was inactivated by adding DMEM/FBS. Cells were then centrifuged at 200 x *g* for 10 minutes. The supernatant was aspirated carefully and the cell pellet was re-suspended in DMEM/FBS. The cell density was adjusted to 8 x 10^4^ cells/mL and each well of a 96-well plate (Cellware) was seeded with 100 μL cell suspension. The medium was aspirated and 100 μL DMEM/FBS with compound or DMSO vehicle was added to the wells. Cells were incubated in a humidified, 37°C, 5% CO_2_ atmosphere for 48 hours. Subsequently, 50 μL pre-mixed Cell-Titer Glo (Promega) was added to each well and the plates were incubated at 22°C for 10 minutes. Luminescence was measured on an Infinite M200 (Tecan). The percent signal was calculated by dividing the luminescent signal from a compound treated well by a vehicle treated well and multiplying by 100. The CC_50_ was calculated by plotting the percent signal vs. compound concentration and fitting to the equation: Percent signal = Min + (Max - Min)/(1 + 10ˆ((Log (IC_50_) ‒ Log (Concentration)) x (Hill Slope))) using GraphPad Prism.

#### Compound stability

Compounds at a final concentration of 10 μM were incubated in modified Roisin’s medium at pH 5.0 or pH 6.6 for 0, 1, 2, or 3 weeks at 37°C. Samples were analyzed by liquid chromatography mass spectrometry (University of Illinois at Urbana-Champaign Mass Spectrometry Lab). Briefly, acetonitrile and formic acid were added to samples to final concentrations of 50% (v/v) and 0.1% (v/v), respectively. The MS analysis was conducted on an LTQ XL Orbitrap mass spectrometer (Thermo Fisher Scientific) operated in positive mode at a resolution of 30000. The spray voltage was 5.0 kV and the capillary temperature was 275°C. The capillary voltage was 23 V. The m/z peaks corresponding to [M + H]^+^, [M + Na]^+^, and [M + K]^+^, where M is a compound of interest, were calculated and manually assigned with the aid of ChemDraw (PerkinElmer). The ion count from each assigned m/z peak was used to monitor relative compound concentration over time.

#### Enumeration of colony forming units (CFU)

*Mtb* was cultured in 96-well plates as above for MIC determination in either pH 6.6 or pH 5.0 modified Roisin's medium. Separate plates were incubated for 0, 7, 14, or 21 days in a humidified environment at 37°C and 5% CO_2_. Where noted, hypoxia was achieved by chemical depletion using a type A Bio-Bag environmental chamber (BD) according to the manufacturer's instructions. The chamber is reported to reach <1% oxygen within 4 hours and a resazurin indicator remained colorless within the Bio-Bag, confirming that the chamber remained hypoxic during the course of the experiment. At each time point, 10-fold serial dilutions (1 x 10^-1^, 1 x 10^-2^, 1 x 10^-3^, 1 x 10^-4^ or 1 x 10^-2^, 1 x 10^-3^, 1 x 10^-4^, 1 x 10^-5^) from each well were plated on 7H11 Middlebrook agar with 10% OADC and 0.5% glycerol. CFU were enumerated after 3-4 weeks incubation at 37°C, and 5% CO_2_.

#### Selection for resistant mutants

Roisin’s solid medium was generated by adding 10 g/L bacterial agar (BD) to modified Roisin’s medium. Compounds were added to the agar to a final concentration of 5x or 10x MIC (for the latter, this corresponds to 60 μM AA691, 120 uM AA692, or 0.8 μM isoniazid). The final concentration of DMSO was 0.5% (v/v) for all plates. *Mtb* H37Rv was grown to an OD_600_ 0.6-0.8 in modified Roisin’s medium and an estimated 4 x 10^7^ and 4 x 10^8^ CFU were plated based on the estimate that OD_600_ 1 is approximately 3 x 10^8^ CFU/mL. Plates were incubated for 5-6 weeks in a humidified environment at 37°C, 5% CO_2._ The number of cells plated in each experiment was confirmed by plating 10-fold serial dilutions on Roisin’s solid medium containing DMSO vehicle control. Two biological replicates were performed and yielded similar results.

#### ABPP-SILAC for competitive ABPP at pH 5.0 and for detection of the active serine hydrolase proteome

Competitive ABPP-SILAC at pH 5.0 was performed as described for target identification by ABPP-SILAC but with the following changes. After growth to OD_600_ ∼1 in light or heavy modified Roisin’s medium at pH 6.6, cells were pelleted, washed twice with modified Roisin’s medium at pH 5.0, and incubated in light or heavy modified Roisin’s medium at pH 5.0 for 3 days. Following this adaptation period, light cultures were incubated with 13 μM AA691, AA692, AA701 or AA702 based on the approximate MIC_90_ for AA692 (Table 1). To identify the total active serine hydrolase proteome, ABPP-SILAC was performed as described above except that light lysates were treated with 0.4% (v/v) DMSO vehicle control and heavy lysates were treated with 4 μM FP-biotin (gift of Dr. Eranthie Weerapana) (Liu et al., 1999).

#### ABPP-SILAC mass spectrometry sample preparation and data analysis

Biotinylated proteins were enriched by incubating combined light/heavy lysates with 100 μL streptavidin agarose beads (Sigma-Aldrich) with gentle shaking at 25°C for 2 hours. After removing the supernatant, the beads were washed three times with 0.25% SDS in 1 mL PBS, once with 1 mL PBS, and once with 1 mL deionized water for a total of five washes. The beads were then resuspended in 500 μL 6 M urea in PBS and treated with 25 μL 200 mM dithiothreitol in water for 15 min at 65°C. The beads were then treated with 25 μL 400 mM iodoacetamide in water for 30 min at 37°C. Samples were diluted with 950 μL PBS to stop the reaction, beads were pelleted at 1400 x *g* for 3 minutes, and the supernatant was aspirated. On-bead protease digestion was performed using 2 mg sequence-grade trypsin (Promega) in 2 M urea and 2 mM CaCl_2_ in PBS for 12-14 hours at 37°C. Peptides released by digestion were acidified with 5% formic acid and stored at -20°C prior to analysis. Digested peptides were analyzed as described previously (Hsu et al., 2012). Briefly, peptides were loaded onto a biphasic (strong cation exchange/reverse phase) capillary column and analyzed by multidimensional liquid chromatography tandem mass spectrometry (MudPIT) LC-MS/MS on an LTQ-Orbitrap (Thermo Scientific). Peptides were eluted using standard gradients and instrument methods, for example: 0%, 25%, 50%, 80%, and 100% salt bumps of 500 mM aqueous ammonium acetate. Data were collected in data-dependent acquisition mode with dynamic exclusion turned on (20 s, repeat of 1). Specifically, one full MS (MS1) scan (400-1800 m/z) was followed by 30 MS2 scans of the most abundant ions. The MS2 spectra data were extracted from the raw file using RAWXtract 1.9.9.2 (McDonald et al., 2004). The ProLuCID algorithm (Xu et al., 2015) was used to search spectra against a *Mtb* H37Rv reverse-concatenated nonredundant (gene-centric) FASTA database that was assembled from the UniProt database. SILAC ratios were quantified using in-house CIMAGE software (Weerapana et al., 2010).

For further analysis and comparison, SILAC ratios were converted to percent inhibition values. Note that all inhibition ratios >20 were reported as 20 and thus the maximum percent inhibition was 95. For the purposes of this study, negative percent inhibition (SILAC ratios >1) was treated numerically as zero inhibition. Under replicating conditions (pH 6.6), serine hydrolases that were inhibited 13% more by AA692 than AA702 (half a standard deviation greater than the mean of all inhibition values measured) were considered prioritized targets (Table S5). Using an analogous cutoff but under non-replicating conditions (pH 5.0), serine hydrolases that were inhibited 36% more by AA692 than AA702 were considered prioritized targets.

#### Enzyme purification

The plasmid pET-15b_6His_Ag85A (BEI, NR-13292) encodes for Ag85A (Rv3804c), hereafter referred to as FbpA. This plasmid was transformed into *E. coli* BL21(DE3) cells and selected on LB plates containing 100 μg/mL carbenicillin (LB/carb100). A single carbenicillin resistant colony was grown in LB/carb100 overnight. This culture was used to inoculate 1 L of LB/carb100 and grown at 37°C until OD_600_ 0.6-0.8 was reached. Expression of FbpA was induced by 500 μM IPTG and the culture was grown at 18°C for 16 hours. After induction, all purification steps were performed at 4°C. Cells were centrifuged at 5000 x *g* for 20 min and the supernatant was removed. Cell pellets were resuspended in 30 mL lysis buffer (20 mM Tris, 200 mM NaCl, 1 mM DTT, 0.2 mM EDTA, 10 mM imidazole, 10% glycerol, pH 7.4) and sonicated with 5 s on/off for 10 min total processing time. The lysate was passed through a 0.45 μm syringe filter before loading onto a nickel affinity column (HisTrap FF 5mL, GE Healthcare). The column was washed with 5 column volumes of binding buffer (Buffer A: 50 mM Tris, 1 mM DTT, 10% glycerol, pH 7.4). Bound FbpA eluted at ∼100 mM imidazole over a 0-50% gradient of elution buffer (Buffer A with 1 M imidazole) over 20 column volumes and purity was confirmed by SDS-PAGE.

*Mtb* fatty acid synthase Fas (Rv2524c) was purified as reported (Baron et al., 2018) from *E. coli* BL21(DE3) harboring the plasmids pMT100-Strep-Flag-*fas1* and pACYCDuet-Ara-*acpS* except that the cell lysate was loaded directly onto a 5 mL Strep-Trap HP column (GE Healthcare) without ammonium sulfate precipitation. Briefly, cells were grown at 37°C in LB broth containing 100 μg/ml ampicillin and 17 μg/ml chloramphenicol to OD_600_ 0.3-0.4. Expression of AcpS was induced by addition of 0.2% arabinose and the culture was grown to OD_600_ 0.6-0.8. The expression of Fas was induced by addition of 0.5 mM IPTG at 15°C for 20 hrs. The cells were harvested and resuspended in buffer A (100mM potassium phosphate pH 7.2, 150 mM KCl, 1 mM TCEP, 1 mM EDTA). After loading onto the Strep-Trap HP column, the column was washed with buffer A and eluted with buffer A containing 2.5 mM desthiobiotin. The thioesterase TesA (Rv2928) and the monoacylglycerol lipase Rv0183 were expressed and purified as reported (Cotes et al., 2007; Nguyen et al., 2018b). Briefly, for TesA, *E. coli* T7 Iq pLysS cells (New England Biolabs) harboring the plasmid pDEST14-TesA were grown in Terrific Broth to OD_600_ 0.6–1.0 induced by with 0.5 mM IPTG overnight at 17°C. Following cell harvesting and lysis by sonication in lysis buffer [50 mM Tris–HCl (pH 8), 300 mM NaCl, 10 mM imidazole, 0.25 mg/mL lysozyme], the supernatant was loaded onto HisTrap 5 mL (GE Healthcare). The protein was washed with buffer A [20 mM Tris–HCl (pH 8), 150 mM NaCl] containing 50 mM imidazole and eluted with buffer A containing 250 mM imidazole. Rv0183 was purified similarly, except *E. coli* Rosetta pLysS cells harboring pDEST14-His-Rv0183 were induced with 1 mM IPTG at 25°C overnight and the lysis buffer was 50 mM Tris/HCl (pH 8.0), 150 mM NaCl, 1 mM EDTA, 0.1% Triton X-100, 0.25 mg/ml lysozyme.

#### *In vitro* competitive ABPP assay

Purified proteins [Fas in 100 mM potassium phosphate buffer, pH 7.4; TesA or FbpA in 20 mM Tris-HCl, pH 7.4, 150 mM NaCl, 0.5% (w/v) Triton X-100] were incubated at 0.1 μM with various concentrations of compound for 2 hours at 22°C. For TesA the catalytically inactive mutant S104A (Nguyen et al., 2018b) was also included as a negative control. Appropriate mutants were not readily available for FbpA and Fas. For these enzymes, heat-treated samples (10 min at 93°C) were included as a negative control. Subsequently, 5 μM TAMRA-FP (gift of Dr. Micah Niphakis) (Patricelli et al., 2001) was added and incubated for 1 hour at 22°C. The final DMSO concentration was 2.5% (v/v). The reactions were quenched with the addition of 5x Laemmli sample buffer, separated by SDS-PAGE (10% for Fas; 15% for TesA, FbpA), and imaged with a Sapphire Biomolecular Imager (Azure) at 520 nm excitation wavelength and 575 nm emission wavelength. All image processing and analysis were performed using Image Studio Lite (LI-COR). Dose-response curves were fitted in GraphPad Prism8.

#### Inhibition assays on recombinant TesA and Rv0183

The lipase-inhibitor preincubation method was used to test the direct inhibition of TesA or Rv0183 in presence of inhibitors as described previously (Nguyen et al., 2018a; Point et al., 2012). Briefly, an aliquot of each enzyme was pre-incubated at 25°C with each inhibitor (50 μM stock solution in DMSO) at various inhibitor molar excess (*x*_I_) ranging from 0.25 to 300 relative to 1 mol of enzyme. Preincubation with inhibitors was performed in the presence of 0.5% (*w/v*) Triton X-100 for TesA and 3 mM sodium taurodeoxycholate for Rv0183. A sample was collected after 30 min incubation and the residual enzyme activity was measured. The variation in the residual enzyme activity allowed determination of the inhibitor molar excess which reduced the activity to 50% of its initial value (*x*_I50_). In each case, control experiments were performed in the absence of inhibitor. The respective enzymatic activity of TesA was assessed using the *para*-nitrophenyl (pNP) ester release assay with pNP valerate (pNP-C5) as substrate (Nguyen et al., 2018b). Rv0183 residual activity was determined potentiometrically using monoolein as substrate. Dose-response curves were fitted in Kaleidagraph 4.2 (Synergy Software).

#### Construction of Mtb strain overexpressing TesA and susceptibility testing

*Mtb* TesA (*rv2928*) was amplified from *Mtb* H37rv genomic DNA with the forward and reverse primers omlp562 (5′-GCAACAAGATGCATATGATGCTGGCCCGTCACG-3′) and omlp559 (5′-CGACATCGATAAGCTTCTAAGCTCGATCATGCCATTGGAG-3′); restrictions sites are underlined. Using the InFusion system (Takara Bio), TesA was cloned into pRiboI (Van Vlack et al., 2017) using the NdeI and HindII restriction sites to generate the vector pRiboI-TesA. After transformation into *Mtb* to generate the strain *Mtb*::ribo-TesA, single kanamycin-resistant colonies were inoculated in modified Roisin’s medium and grown to OD_600_ 0.3–0.4. Overproduction was induced with the addition of 1 or 2 mM theophylline for 3 days. The MIC of induced TesA overexpressing *Mtb* versus non-induced *Mtb* was measured by visual inspection according to the MIC determination protocol described above.

#### In silico molecular docking experiments

Autodock Vina (Trott and Olson, 2009) was used as previously reported (Nguyen et al., 2017; Seeliger and de Groot, 2010) to generate the putative binding modes of the various inhibitors into the active site of TesA and the Rv0183. The PyMOL Molecular Graphics System (version 1.4, Schrödinger, LLC) was used as working environment with an in-house version of the AutoDock/Vina PyMOL plugin (Seeliger and de Groot, 2010). The X-ray crystallographic structure of TesA in complex with the CyC_17_ inhibitor (PDB: 6FVJ) and Rv0183 (PDB: 6EIC) were used as receptors (Aschauer et al., 2018; Nguyen et al., 2018b). Docking runs were performed after replacing the catalytic serine (*i.e*., Ser104 in TesA and Ser110 in Rv0183) by a glycine residue to enable the ligand (*i.e*., the inhibitor) to adopt a suitable position corresponding to the pre-bound intermediate before the nucleophilic attack in the enzyme active site. The box size used for the various receptors was chosen to fit the whole enzyme’s active site cleft and allowed non-constructive binding positions. The three-dimensional structures of the aforementioned compounds were constructed using Chem3D Ultra 11.0 software, and their geometry was refined using the Avogadro 1.2.0 open-source molecular builder and visualization tool (Hanwell et al., 2012).

### Quantification and statistical analysis

Statistical details of experiments, including number of independent experiments and/or replicates used for analysis; data reported; and statistical tests used, are provided in the figure legends. In general significance was determined as p < 0.05 (values for specific comparisons are also noted in the figure legends). Statistical analyses were performed with GraphPad Prism 8.

## Data Availability

Proteomics datasets from this study are deposited at PRIDE. MorphEUS analysis data from this study have been deposited at GitLab and are publicly available as of the date of publication. DOIs are listed in the key resources table. This paper does not report original code. Any additional information requires to reanalyze the data reported in this paper is available from the lead contct upon request.

## Supplemental information

Document S1. Figures S1–S7

Table S1. Summary of ABPP-SILAC experiments, related to Table 2

Table S2. Peptides detected in ABPP-SILAC experiments, related to Table 2

Table S3. Active serine hydrolase proteome, related to Table 2

Table S4. Prioritized serine hydrolase targets of AA692, related to Table 2

Table S5. Visual MIC for Mtb::ribo-TesA, related to Table 2

Document S2. Article plus supplemental information
