# Supplementary material for: Identification of cell wall synthesis inhibitors active against Mycobacterium tuberculosis by competitive activity-based protein profiling
Source: Cell Chem Biol. 2022 May 19;29(5):883–896.e5. doi: 10.1016/j.chembiol.2021.09.002 (PMC8964833; doi:10.1016/j.chembiol.2021.09.002)
Supplement: Document S1. Figures S1–S7 [file mmc1.pdf]

Cell Chemical Biology, Volume *29*

Supplemental information

Identification of cell wall synthesis inhibitors active against *Mycobacterium tuberculosis* by competitive activity-based protein profiling

Michael Li, Hiren V. Patel, Armand B. Cognetta III, Trever C. Smith II, Ivy Mallick, Jean-François Cavalier, Mary L. Previti, Stéphane Canaan, Bree B. Aldridge, Benjamin F. Cravatt, and Jessica C. Seeliger

SUPPLEMENTAL FIGURES

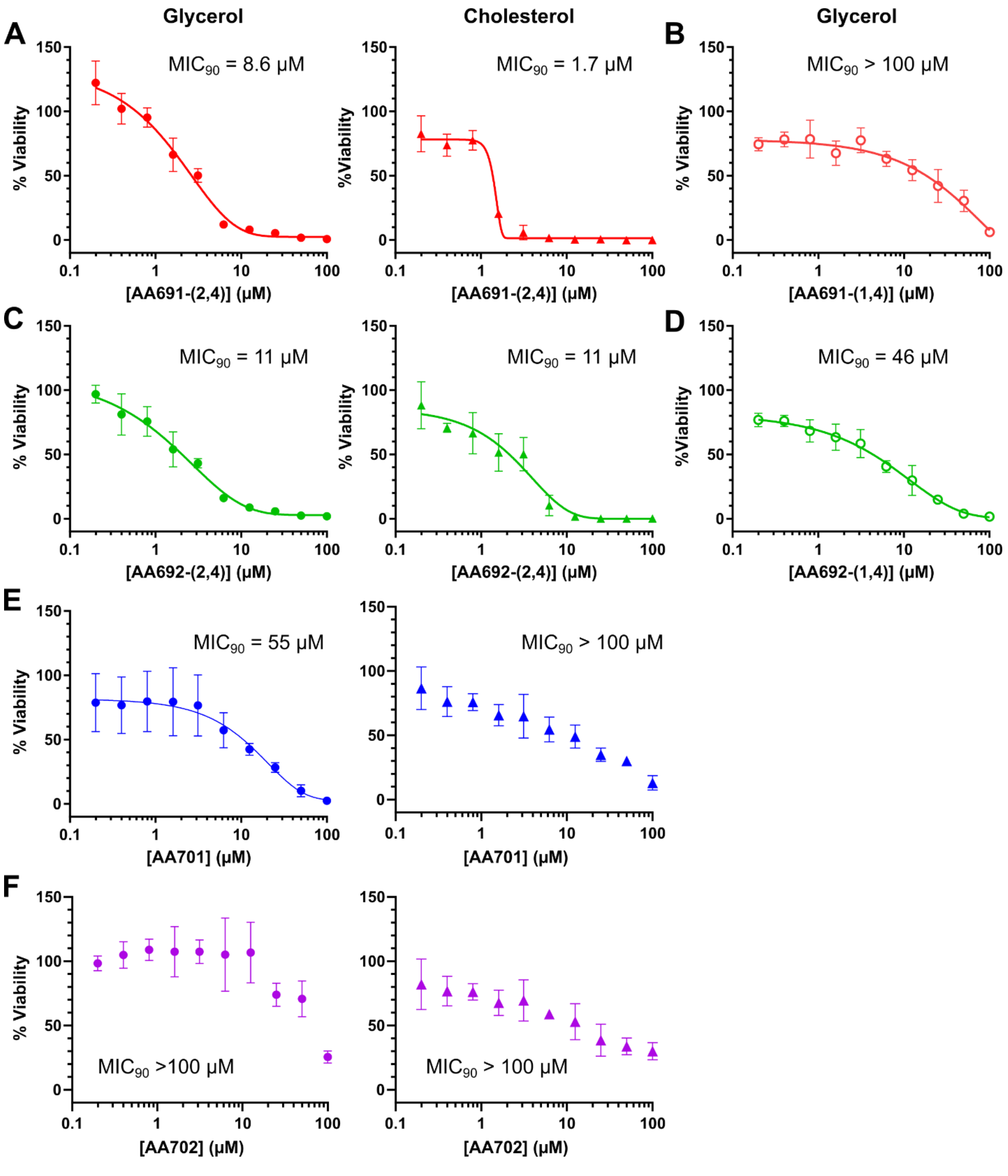

Figure S1. Dose response curves confirm structure-activity relationships under replicating conditions and AA691 and AA692 are active against pH-induced non-replicating Mtb, Related to Table 1. Autoluminescent *Mtb* was treated with A) AA691, B) AA691-(1,4), C) AA692, D) AA692-(1,4), E) AA701, or F) AA702 in modified Roisin's medium containing glycerol or cholesterol as indicated. Data shown are representative of n=1 (B, D) or n=3-5 (A, C, E, F) independent experiments in glycerol and n=2 independent experiments in cholesterol. Each data point is the mean ± S.D. of 3 technical replicates from a single experiment. MICs were determined by fitting the percent viability (versus DMSO vehicle-treated control) to the Gompertz equation.

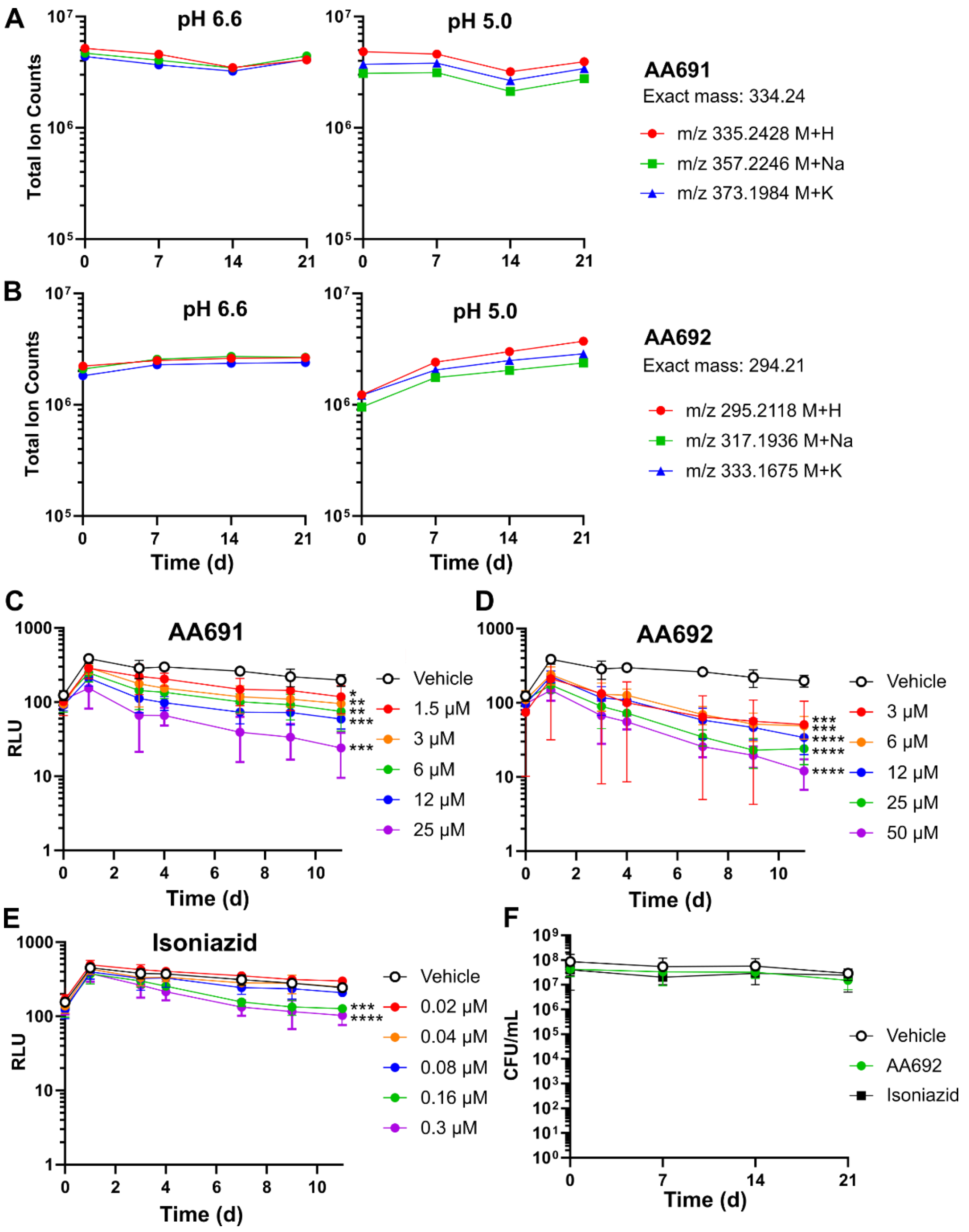

Figure S2. AA691 and AA692 are stable over 3 weeks and active against acidic pH-induced non-replicating *Mtb*, Related to Figure 2. A) AA691 or B) AA692 at a final concentration of 10 μM was incubated in modified Roisin's medium at pH 5.0 or pH 6.6 for 0, 1, 2, or 3 weeks at 37 °C. Samples were analyzed by liquid chromatography mass spectrometry. The *m/z* peaks corresponding to $[M + H]^+$, $[M + Na]^+$, and $[M + K]^+$ were manually assigned. Data are from individual samples at each time point from a single experiment. Autoluminescent *Mtb* were incubated in modified Roisin's medium at pH 5.0 for 3 days before treating with C) AA691, D) AA692, E) isoniazid or F) 12 μM AA692 or 0.08 μM isoniazid for CFU enumeration. * $p < 0.05$, ** $p < 0.005$, *** $p < 0.0005$, **** $p < 0001$ by one-way ANOVA with Dunnett correction for each timepoint vs. vehicle-treated control on day 11. Comparisons at other timepoints were not significant. Data shown are the mean ± S.D. of 3 biological replicates.

A

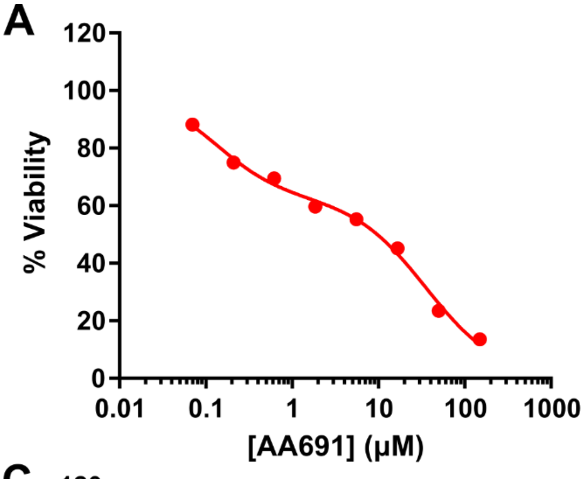

B

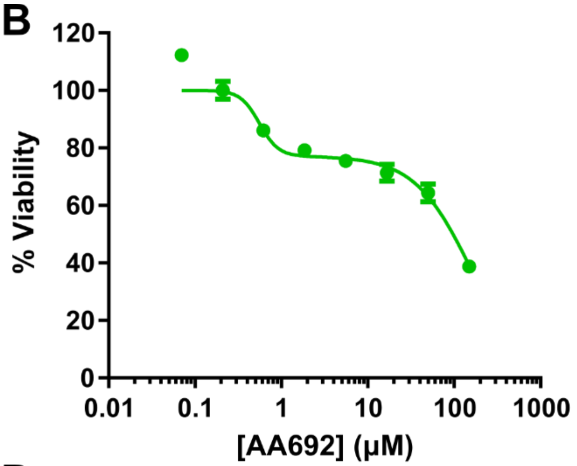

C

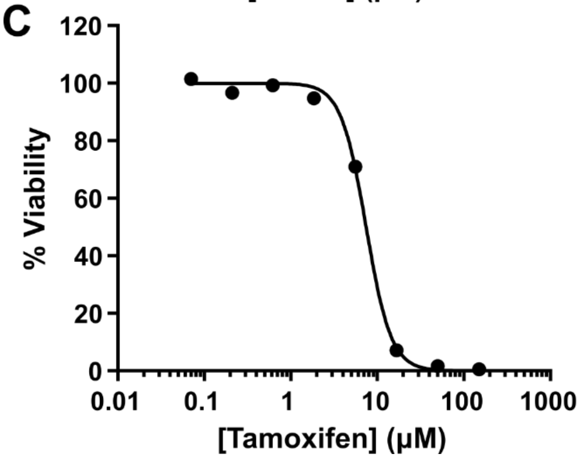

D

Compound	HepG2 CC_{50} (µM)		Mtb MIC (µM)	SI (CC_{50}/MIC)
AA691*	CC_{50}(1)	0.13	7± 4	0.02
	CC_{50}(2)	35		5
AA692	CC_{50}(1)	0.57 ± 0.02	11 ± 4	0.05
	CC_{50}(2)	152 ± 11		14
Tamoxifen		7.4 ± 0.2		
Isoniazid		57,000 **	0.1 ± 0.04	>570,000

Figure S3. AA691 and AA692 are not highly selective for *Mtb*, Related to Figure 2. HepG2 cells were treated with A) AA691, B) AA692, or C) tamoxifen for 48 hours at 37 °C. Cell viability was measured using CellTiter-Glo (Promega). Data shown are the average ± S.D. from n=3 separate experiments. D) The selectivity index (SI) was calculated from the fit CC_{50} and the MICs from Table 1. Data shown are the mean ± S.D. from 3 biological replicates. *S.D. not reported as only 2 of 3 replicates supported a robust biphasic fit. **Elmorsy et al., Adverse effects of anti-tuberculosis drugs on HepG2 cell bioenergetics. *Hum Exp Toxicol*, 2017, 6, 616-625,

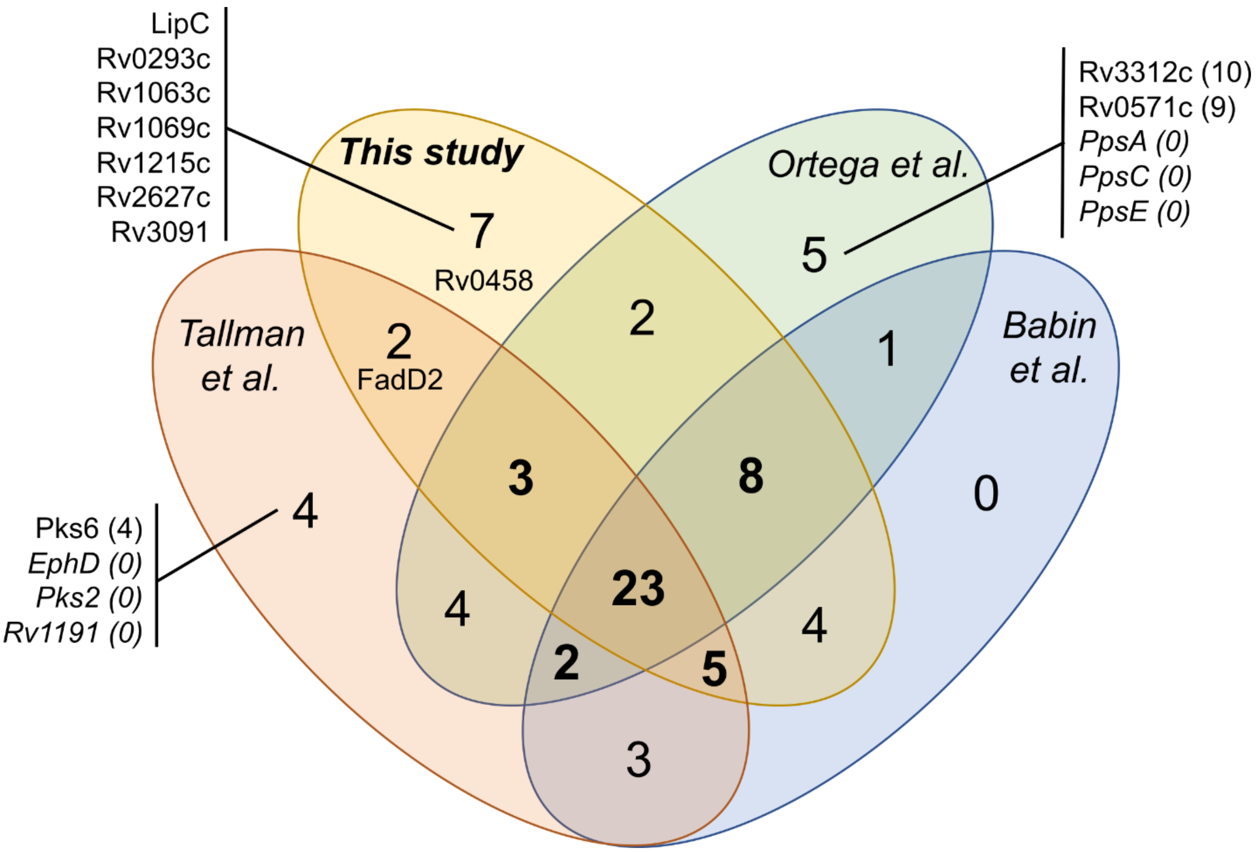

Figure S4. Activity-based protein profiling in *Mtb* by fluorophosphonate probes detects nearly 75 active serine hydrolase across multiple studies, Related to Table 2. The number in parentheses indicates the number of instrumental replicates in which a given serine hydrolase was detected in this study (each biological sample was subjected to 1-2 MS runs; each run was counted as an instrumental replicate). Proteins without this annotation were detected in at least 14 of 15 instrumental replicates and thereby met our cutoff for annotation as a serine hydrolase. Proteins not detected in any instrumental replicates are in italics. The proteins Rv0458 and FadD2 met the detection cutoff, but are unlikely bona fide serine hydrolases and so were not counted. Numbers in bold indicate detection in at least 3 studies.

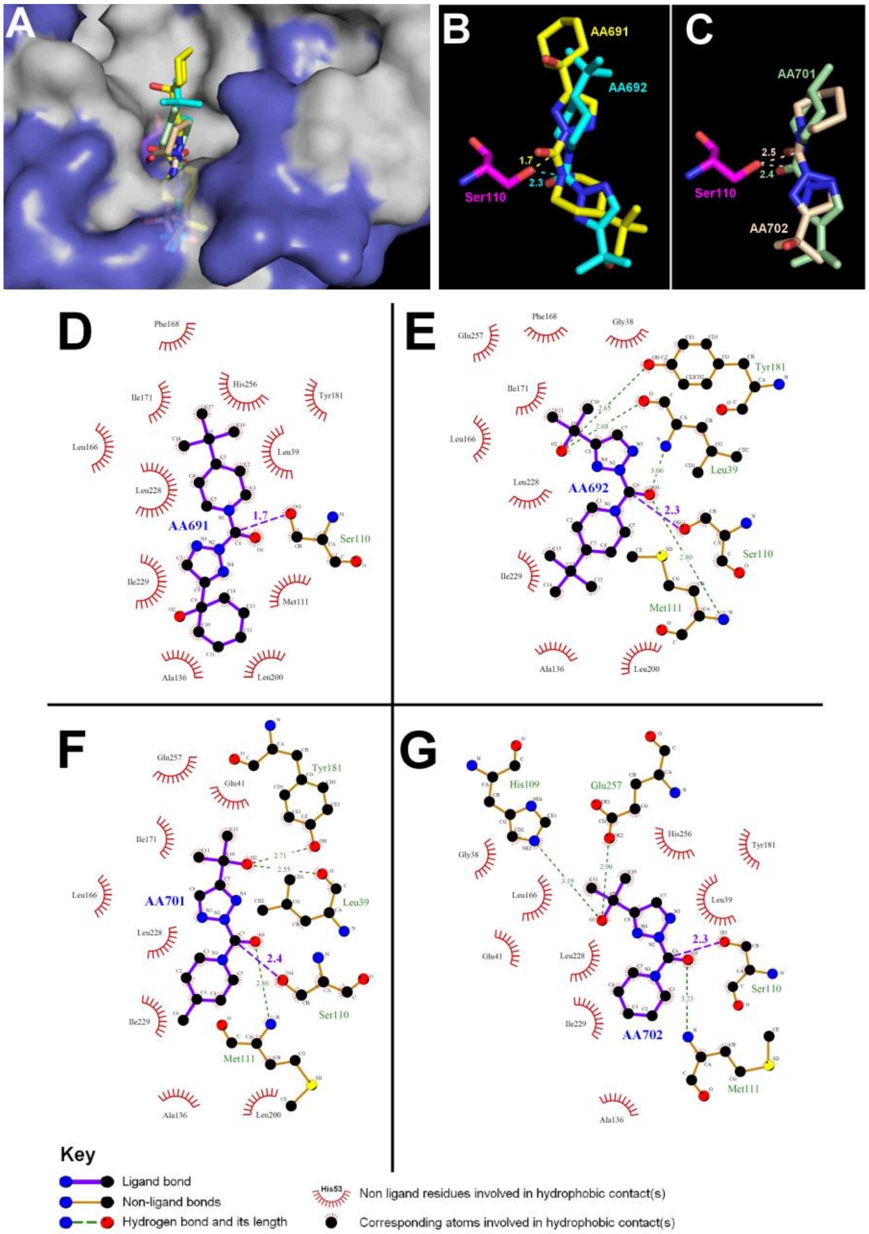

Figure S5. **AA691, AA692, AA701, and AA702 adopt similar poses in the Rv0183 active site, Related to Figure 4.** A) *In silico* molecular docking of AA691, AA692, AA701 and AA702 into the crystallographic structure of Rv0183 in a van der Waals surface representation. Hydrophobic residues are highlighted in *white*. Superimposition of the top-scoring docking position of B) AA691 (*yellow*) and AA692 (*cyan*) and C) AA701 (*palegreen*) and AA702 (*wheat*) in the vicinity of the catalytic Ser110 (*magenta*). Structures were drawn with PyMOL using the PDB file 6EIC. Ligplot^{+} (Laskowski and Swindells, 2011) analyses showing ligand-protein interactions for D) AA691; E) AA692; F) AA701 and H) AA702 in the Rv0183 active site with hydrogen bonds (*purple, green* dashed lines) and hydrophobic interactions (*red*) indicated.

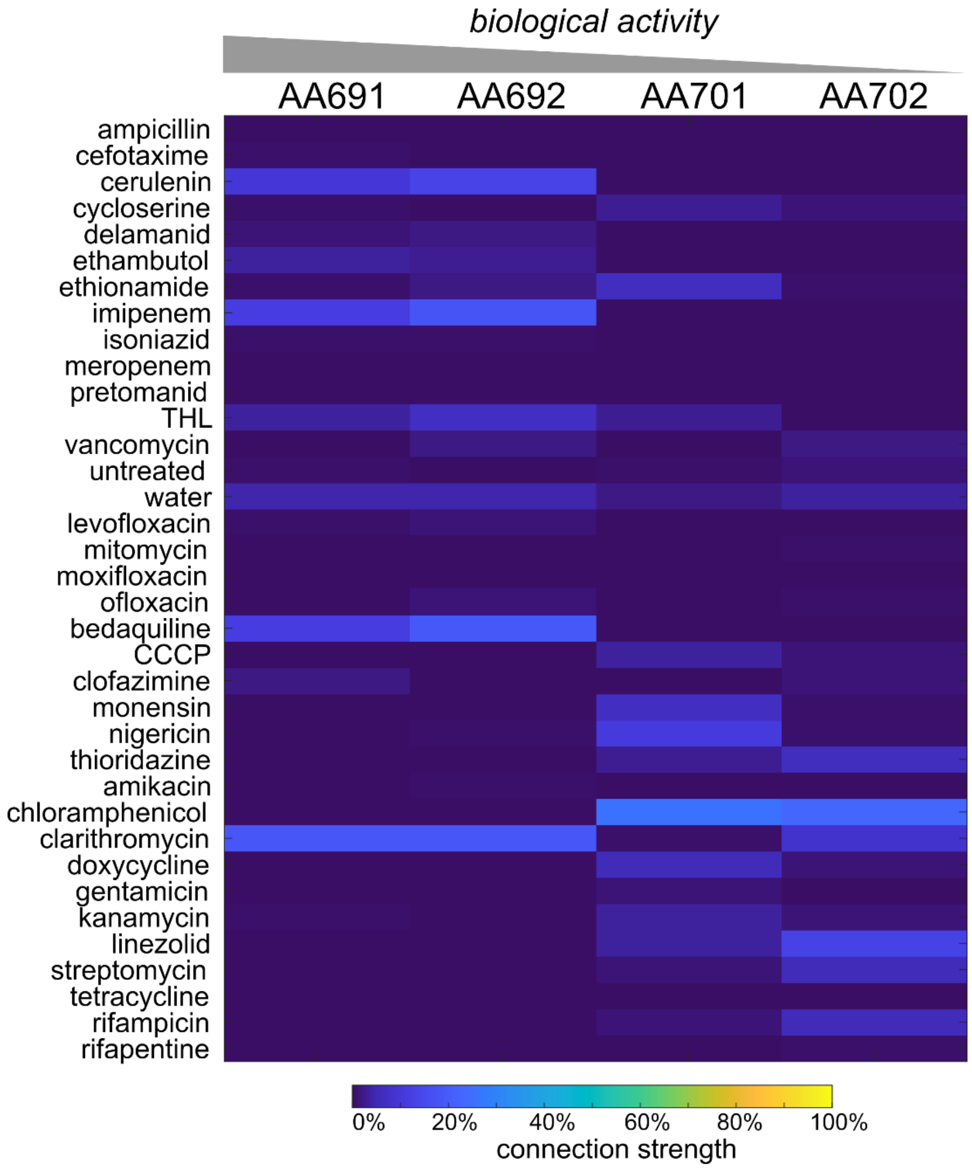

Figure S6. AA691 and AA692 at low dose cause morphological changes in *Mtb* that are similar to cell wall synthesis inhibitors and to the translation inhibitor clarithromycin, Related to Figure 5. *Mtb* were incubated with 50 μM compound (low dose) and stained for cellular membranes and the chromosomal nucleoid. Stained *Mtb* were imaged and analyzed for 25 morphological features. The profile for each compound was applied onto the morphological space constructed using 34 compounds with known mechanisms of action. The resulting nearest neighbor frequency (connection strength) based individual compounds is highest among drugs that cause similar types of cellular change.

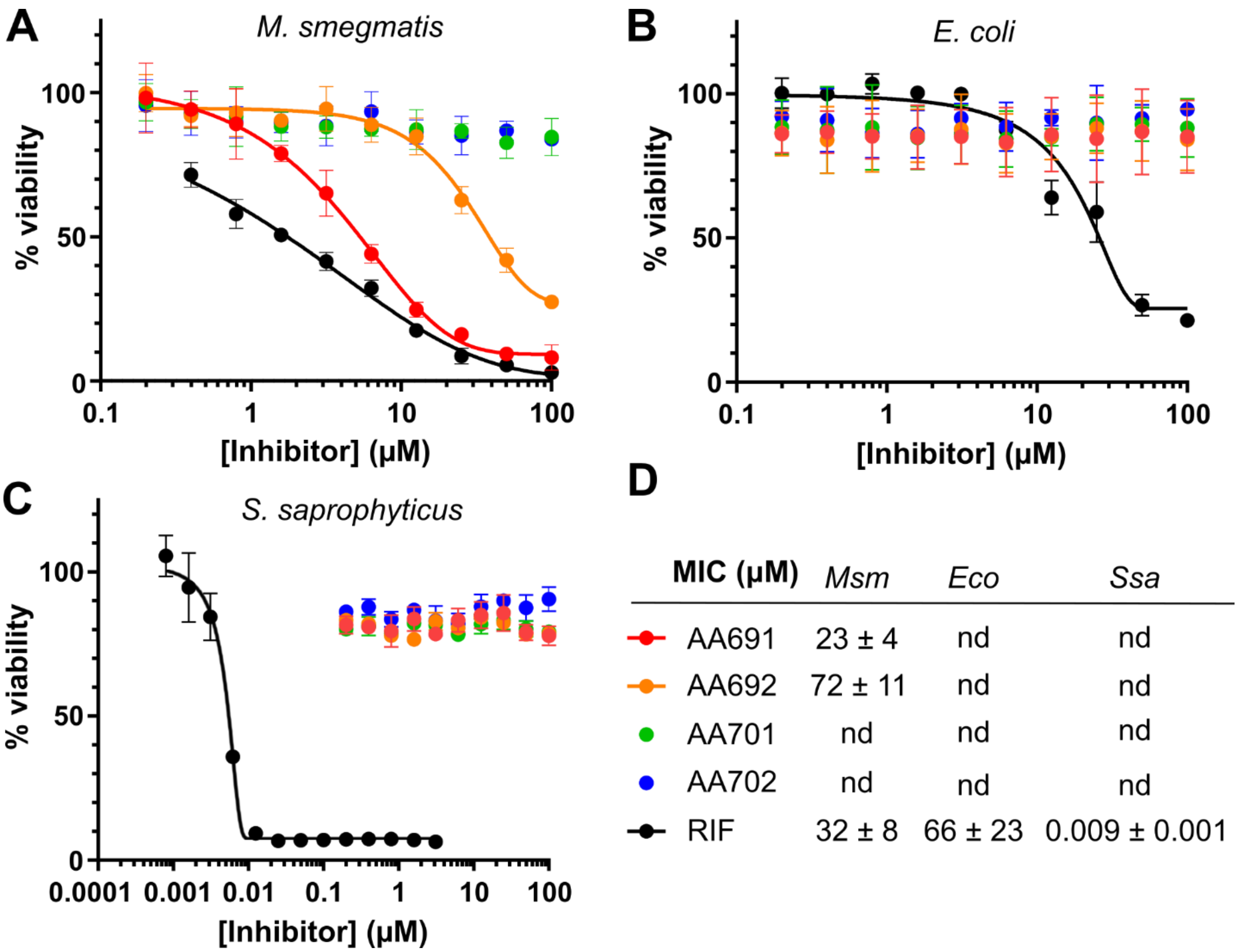

D

MIC (μM)	*Msm*	*Eco*	*Ssa*
AA691	23 ± 4	nd	nd
AA692	72 ± 11	nd	nd
AA701	nd	nd	nd
AA702	nd	nd	nd
RIF	32 ± 8	66 ± 23	0.009 ± 0.001

Figure S7. AA691 and AA692 have narrow-spectrum antibiotic activity, Related to Table 1. A) Autoluminescent *M. smegmatis,* B) *E. coli* and C) *S. saprophyticus* were treated with the indicated compounds for ~3 doubling times for each respective bacterium. The viability of *was* determined by autoluminescence for *M. smegmatis* and by Bac-Titer Glo for *E. coli* and *S. saprophyticus*. Data shown are the mean ± S.D. of 3 technical replicates from one representative experiment in A)-C). MICs were determined by fitting the percent inhibition (versus DMSO vehicle-treated control) to the Gompertz equation and the mean ± S.D. from 3 independent experiments is reported in D); nd, not detected.
